# Distinct breast cancer stem/progenitor cell populations require either HIF1α or loss of PHD3 to expand under hypoxic conditions

**DOI:** 10.18632/oncotarget.5564

**Published:** 2015-09-10

**Authors:** Oihana Iriondo, Miriam Rábano, Giacomo Domenici, Onintza Carlevaris, José Antonio López-Ruiz, Ignacio Zabalza, Edurne Berra, Maria dM Vivanco

**Affiliations:** ^1^ Cell Biology and Stem Cells Unit, CIC bioGUNE, Derio, Spain; ^2^ Servicio de Radiodiagnóstico Preteimagen, Bilbao, Spain; ^3^ Department of Pathology, Galdakao-Usansolo Hospital, Galdakao, Spain

**Keywords:** breast cancer, estrogen receptor, hypoxia, PHD3, stem cells

## Abstract

The heterogeneous nature of breast cancer is a result of intrinsic tumor complexity and also of the tumor microenvironment, which is known to be hypoxic. We found that hypoxia expands different breast stem/progenitor cell populations (cells with increased aldehyde dehydrogenase activity (Aldefluor^+^), high mammosphere formation capacity and CD44^+^CD24^−/low^ cells) both in primary normal epithelial and tumor cells. The presence of the estrogen receptor (ER) limits hypoxia-dependent CD44^+^CD24^−/low^ cell expansion. We further show that the hypoxia-driven cancer stem-like cell enrichment results from a dedifferentiation process. The enhanced mammosphere formation and Aldefluor^+^ cell content observed in breast cancer cells relies on hypoxia-inducible factor 1α (HIF1α). In contrast, the CD44^+^CD24^−/low^ population expansion is HIF1α independent and requires prolyl hydroxylase 3 (PHD3) downregulation, which mimics hypoxic conditions, leading to reduced CD24 expression through activation of NFkB signaling. These studies show that hypoxic conditions expand CSC populations through distinct molecular mechanisms. Thus, potential therapies that combine current treatments for breast cancer with drugs that target CSC should take into account the heterogeneity of the CSC subpopulations.

## INTRODUCTION

Breast cancer, the most frequent malignancy in the female population in incidence and mortality [1], is a very heterogeneous disease in terms of histology, genetic profile, therapeutic response and patient outcome. Global gene expression studies of breast tumors led to the description of five different breast cancer subtypes -luminal A, luminal B, HER2-positive, basal and normal-like- with distinct clinical outcomes [2-4] and a novel molecular stratification was derived from the association of somatic copy number aberrations with the transcriptome [5, 6], highlighting the molecular heterogeneity of the disease. Approximately 70% of breast tumors express the estrogen receptor (ER) and, in general, ER expression is associated with better prognosis [7].

The tissue expansion and remodeling that occurs in the mammary gland during successive cycles of pregnancy, lactation and involution has been linked to the presence of stem cells and early progenitor cells in the adult mammary epithelium [8]. Similarly, breast tumors are also composed of morphologically and phenotypically heterogeneous cell populations, characterized by varying self-renewal capacities, degrees of differentiation and tumorigenic potentials. In the apex of the hierarchy lie the cancer stem cells (CSCs), also known as tumor initiating cells. In addition to directing tumor onset and metastatic expansion, CSCs have been shown to be resistant to chemo- and radiotherapy and more recently also to endocrine therapy [9], and could therefore be responsible for tumor recurrence. Several methods have been used to identify and isolate human breast epithelial stem cells and cancer stem cells. In the normal breast, cells coexpressing the luminal marker EMA and the myoepithelial marker CALLA [10], CD49f^high^ESA^−/low^ cells [11, 12], and cells with high ALDH activity [13], have been shown to be enriched in bipotent progenitors. Furthermore, the phenotype CD44^+^CD24^−/low^ESA^+^ and high ALDH activity identify cells with increased tumor initiation capacity [13, 14]. Importantly, CD44^+^CD24^−/low^ESA^+^ cells, ALDH^+^ cells and mammosphere-forming cells isolated from breast cancer cell lines are also enriched for self-renewal capacity and tumorigenic potential in xenograft tumor assays [15, 16].

In solid tumors, the combination of rapid cell division and aberrant tumor angiogenesis often leads to the generation of hypoxic sites [17]. Tumor hypoxia has been associated with increased malignancy, poor prognosis and resistance to radiotherapy and chemotherapy [18]. Hypoxia-inducible factors (HIFs) are the main transcriptional regulators of the adaptive responses that are activated when oxygen supply does not reach the metabolic, energetic and redox demands of cells [19]. In well-oxygenated environments, the HIFα subunit (HIF1α or HIF2α) becomes hydroxylated by members of the prolyl hydroxylase domain-containing proteins (PHD) family (PHD1, PHD2 and PHD3, also known as EGLNs), which use oxygen as co-substrate [20], and is targeted for degradation by the proteasome [21, 22]. Under low oxygen availability, PHDs are inactive, HIFα is stabilized, dimerises with HIFβ and regulates the transcription of target genes [23]. Although the role of PHDs in cancer has been less studied, altered levels of PHD1, PHD2 and PHD3 have been correlated with the development of different types of carcinomas [24-26]. Distinct members of the hypoxia-signaling pathway are involved in the regulation of both normal and cancer stem cells. In breast cancer cells, antiangiogenic factors increase the population of CSCs by generating intratumoral hypoxia mediated by HIF1α [27]. Furthermore, HIF factors have also recently been implicated in the enhancement of breast CSCs by chemotherapy [28].

Considering the links of hypoxia with cancer and stem cells, we wished to investigate the molecular mechanisms that underlie the impact of hypoxic conditions on the cancer stem cell compartment. Here, we show that hypoxia increases the proportion of breast CSCs through a dedifferentiation process and limits the differentiation of CSCs. Depending on the stem/progenitor cell subpopulation, this process requires either HIF1α expression or the inactivation of the hydroxylase activity of PHD3. These findings suggest that different therapeutic strategies should be adopted to eliminate hypoxia-induced breast cancer stem cells depending on tumor characteristics.

## RESULTS

### Hypoxia increases the proportion of primary breast stem cells and tumor initiating cells

**Figure 1:**
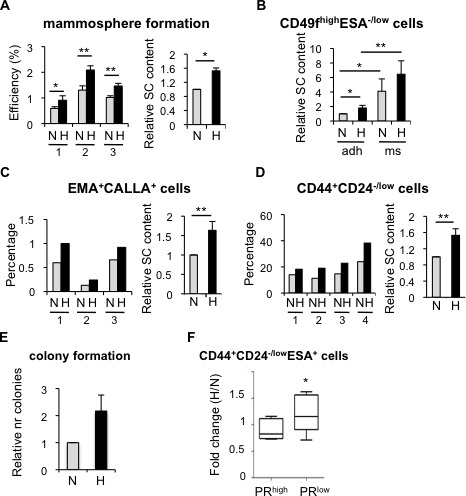
Effect of hypoxia in cells isolated from normal or tumor primary tissue **A.** Normal primary epithelial cells cultured in suspension in normoxia or hypoxia for 7 days. Mammosphere formation efficiency of cells from four different breast specimens (left graph) and mean ±SD of three experiments (right graph) are represented as the percentage of mammospheres formed with respect to the number of plated cells, and as fold change between normoxia and hypoxia, respectively. **B.** Percentage of CD49f^high^ESA^−/low^cells in normal breast epithelial cells cultured in normoxia or hypoxia, in adherent (adh) or suspension (ms) conditions. **C.**, **D.** Percentage of EMA^+^CALLA^+^ and CD44^+^CD24^−/low^ cells in normal breast epithelial cells cultured in normoxia or hypoxia. **E.** Relative number of colonies formed in Matrigel by primary normal epithelial cells cultured in normoxic or hypoxic conditions. **F.** Percentage of CD44^+^CD24^−/low^ESA^+^ cells found in primary tumor cells cultured as mammospheres in normoxic or hypoxic conditions and represented as fold change between hypoxia and normoxia. The graph shows the mean ±SD of the fold changes grouped based on high or low ER transcriptional activity (PR^high^ or PR^low^, respectively, low was defined as less than 11% expression) *P < 0.05 (*P* = 0.031).

Firstly, to investigate whether hypoxia had any effect in the pool of normal stem/progenitor cells of the human mammary gland, different stem cell subpopulations were examined. Cells are routinely cultured in atmospheric oxygen (21% O_2_), although this is not physiological. Normal pO_2_ in the breast is 8.6% [29], nevertheless, comparable CSC activity has been reported between 21% and 8% oxygen [30]. Hypoxic conditions are usually represented as 1% O_2_, despite the fact that the average pO_2_ in breast cancer is 3.9%, although in approximately 30-40% of the cases tumors exhibit pO_2_ values between 0 and 1% [29]. Thus, breast epithelial cells isolated from reduction mammoplasties (Supplementary Table 1) were cultured in suspension in atmospheric oxygen, which will be referred to as normoxic (21% O_2_) or under hypoxic conditions (1% O_2_). After 7 days, cells grown in hypoxia formed more mammospheres, which are enriched for stem/progenitor cells, than cells cultured in atmospheric oxygen concentration (Figure 1A). Furthermore, cells cultured under hypoxia were enriched in CD49f^high^ESA^−/low^ cells (Figure 1B; Supplementary Figure 1A, 1B), independently of whether cells were cultured in adherent or suspension conditions. Similarly, EMA^+^CALLA^+^ (Figure 1C; Supplementary Figure 1C) and CD44^+^CD24^−/low^ (Figure 1D; Supplementary Figure 1D) stem cell subpopulations were also enhanced under hypoxia. Moreover, hypoxic conditions increased the ability of primary breast epithelial cells to form colonies in Matrigel at low density (Figure 1E), suggesting that decreased oxygen availability leads to the expansion of the pool of stem/progenitor cells in the normal mammary gland.

To evaluate whether hypoxia also influences the proportion of CSCs, tumor cells isolated from breast cancer patients were grown in suspension in normoxic or hypoxic culture conditions. The effect of hypoxia on breast CSCs was tumor-dependent. The proportion of CD44^+^CD24^−/low^ cells was not significantly affected by hypoxia in those samples that presented high levels of ER and PR expression (Figure 1F, PR^high^). In contrast, in tumor samples lacking ER expression or with low ER transcriptional activity (as reflected by low PR expression, PR^low^), hypoxia promoted the expansion of CD44^+^CD24^−/low^ cells (Figure 1F; Supplementary Figure 1E; Supplementary Table 2). The differences observed in the response to hypoxia likely reflect the high molecular heterogeneity present in breast tumors. Overall these findings suggest that low oxygen availability increases the normal and cancer stem cell content in the breast.

### Hypoxia increases the proportion of cancer stem cells in breast cancer cell lines

**Figure 2:**
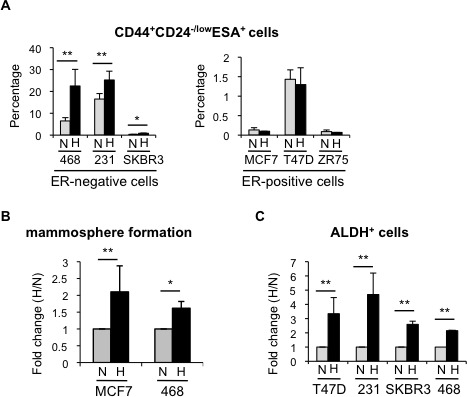
Hypoxia increases the percentage of CSCs in different breast cancer cell lines **A.** Percentage of CD44^+^CD24^−/low^ESA^+^ cells in ER-negative and ER-positive cell lines cultured in normoxia or hypoxia for 3 days. **B.** Number of mammospheres formed by MCF-7 or MDA-MB-468 cells cultured in normoxia or hypoxia and represented as fold change (hypoxia/normoxia). **C.** Percentage of ALDH^+^ cells in different cell lines cultured in normoxia or hypoxia. In A and B, means ±SD of at least three independent experiments are represented. *P < 0.05 **P < 0.01.

In order to investigate how hypoxic conditions influence breast CSCs and the mechanisms implicated, we examined the effects of hypoxia in several breast cancer cell lines. Firstly, using MDA-MB-468 cells, we observed a significant increase in CD44^+^CD24^−/low^ESA^+^ cells, which reached a plateau by 48-72 hours treatment (Supplementary Figure 2A) and, therefore, we evaluated the effect of 3-day long hypoxia treatment on the CSC populations in a panel of ER-positive and ER-negative breast cancer cell lines. FACS analysis showed that ER-negative MDA-MB-468, MDA-MB-231 and SKBR3 cells cultured in hypoxic conditions contained a higher proportion of CD44^+^CD24^−/low^ESA^+^ cells than their normoxic counterparts. In contrast, the CD44^+^CD24^−/low^ESA^+^ content of ER-positive MCF-7, T47D and ZR75-1 cells was not significantly affected by hypoxia (Figure 2A; Supplementary Figure 2B). The observed expansion of CD44^+^CD24^−/low^ESA^+^ cells by hypoxia encouraged us to examine whether oxygen levels affected the proportion of different subpopulations of CSCs in breast cancer cells. Hypoxic conditions increased the mammosphere forming capacity of both ER-positive (MCF-7) and ER-negative (MDA-MB-468) cells (Figure 2B; Supplementary Figure 2C). Furthermore, a cell population with ALDH activity, as measured by ALDEFLUOR assay, ALDH^+^, was also increased in response to hypoxia in both ER-positive and ER-negative cells (Figure 2C; Supplementary Figure 2D). These findings indicate that hypoxic conditions lead to expansion of different types of CSC subpopulations and that the levels of ER expression in breast cancer cells may influence their response.

### Hypoxia reduces ER expression and transcriptional activity

**Figure 3:**
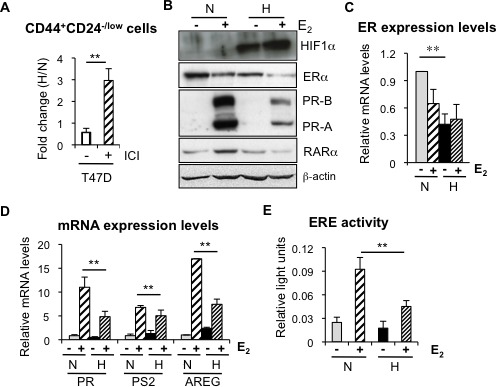
Hypoxia reduces ER expression and transcriptional activity **A.** Percentage of CD44^+^CD24^−/low^ cells in T47D cells treated or not with 0,5 μM fulvestrant (ICI 182,870) and cultured in normoxia or hypoxia. **B.** Representative western blot showing expression of ER and its targets PR and RARα in MCF-7 cells cultured under normoxic or hypoxic conditions, with or without 10 nM estrogen (E_2_). **C.** RNA expression levels of ER in MCF-7 cells treated or not with estrogen, in normoxia or hypoxia. **D.** RNA expression levels of PR, PS2 and AREG in MCF-7 cells treated or not with estrogen, in normoxia or hypoxia. In C, D, Data are presented as mean ±SD of 3 independent experiments. **E.** ER transcriptional activity in MCF-7 cells grown in normoxia or hypoxia in the presence of ethanol (−) or estrogen. The graph shows the mean ±SEM of 5 experiments done in triplicates. **P < 0.01.

The above findings suggest that the presence of ER hampers the expansion of CD44^+^CD24^−/low^ cells by hypoxia. To explore this possibility further, ER-positive T47D cells were treated with the ER antagonist fulvestrant (ICI 182,780), leading to strong ER degradation (Supplementary Figure 3A). Indeed, now in the absence of ER, hypoxia induced a significant increase in the percentage of CD44^+^CD24^−/low^ cells in T47D cells (Figure 3A), suggesting that loss of ER is required for hypoxia to expand the CD44^+^CD24^−/low^ cell population.

We have previously shown that normal and CSCs from the mammary gland are characterized by the absence or low expression levels of ER [8]. The finding that ER limits the amplification of CD44^+^CD24^−/low^ CSC subpopulation under low oxygen conditions prompted us to explore this relationship in more detail. To this end, several ER-positive breast cancer cell lines were cultured in the absence or presence of estrogen, in normoxic or hypoxic conditions. Western blot analysis showed that hypoxia treatment reduced ER expression levels in all cell lines tested, MCF7 (Figure 3B), T47D (Supplementary Figure 3A) and ZR75-1 (data not shown). Furthermore, the decrease in ER levels induced by hypoxia was also detected at the RNA level (Figure 3C; Supplementary Figure 3D, 3E). More importantly, the evaluation of the expression levels of several ER target genes (progesterone receptor, retinoic acid receptor alpha, amphiregulin and pS2) (Figure 3B, 3D; Supplementary Figure 3B-3E) and ER activity by transcriptional assays (Figure 3E) clearly showed that hypoxia-dependent decrease in ER expression correlates with reduced estrogen-dependent ER signaling. These findings indicate that hypoxia reduces ER expression and activity in breast cancer cells, thereby enriching for CSCs.

### Hypoxia prevents differentiation of CSCs and promotes dedifferentiation of breast cancer cells

**Figure 4:**
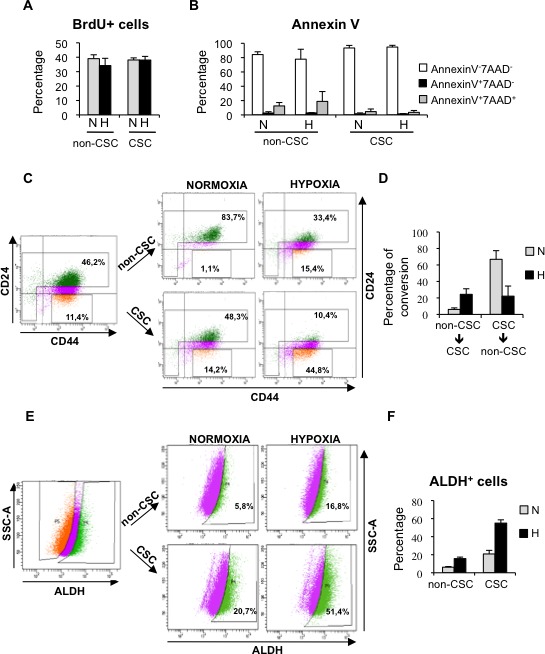
Hypoxia promotes dedifferentiation of breast cancer cells **A.** Percentage of BrdU positive MDA-MB-468 cells in sorted cell populations: CD44^+^CD24^−/low^ CSCs and non-CSCs, grown in normoxia or hypoxia. **B.** Detection of apoptotic cells in CSCs and non-CSCs isolated from MDA-MB-468 cells that were grown in normoxia or hypoxia. The percentages of live cells (AnnexinV^−^7AAD^−^), early apoptotic (AnnexinV^+^7AAD^−^) and late apoptotic (AnnexinV^+^7AAD^+^) cells are shown. **C.** Representative example of a sorting experiment with CD44/CD24-stained MDA-MB-468 cells. On the left, CD44/CD24 staining of MDA-MB-468 cells cultured in hypoxia for 3 days. Gates used to sort CD44^+^CD24^−/low^ cells (CSC) and the cell population depleted of CSCs (non-CSC) are presented. On the right, CD44/CD24 stainings performed with sorted CSCs and non-CSCs after 3 additional days growing in normoxia or hypoxia. **D.** Graph shows the capacity of CD44^+^CD24^−/low^ CSCs to produce CD44^+^CD24^high^ non-CSCs, and vice-versa, in normoxic and hypoxic conditions. **E.** Representative example of a dedifferentiation experiment performed with T47D cells sorted based on their ALDEFLUOR activity. **F.** Percentage of ALDH^+^ cells obtained after culturing ALDH^+^ CSCs and ALDH^−^ non-CSCs in normoxia or hypoxia. A, B, D and F show means ±SD of three independent experiments.

Next, we wished to decipher the process by which hypoxia increases the proportion of CSCs. First, we evaluated whether differences in proliferation or apoptosis between CSC and non-CSCs cultured in normoxic or hypoxic conditions could contribute to the increase in CD44^+^CD24^−/low^ cells observed under low oxygen conditions. BrdU incorporation assay and annexin-V staining showed no significant differences in the response of CSC and non-CSCs to hypoxia in terms of proliferation (Figure 4A; Supplementary Figure 4A) or apoptosis (Figure 4B; Supplementary Figure 4B). To assess further the influence of oxygen concentration in CSC content, MDA-MB-468 cells were cultured in hypoxic conditions for 3 days, in order to enrich for CSCs, and CD44^+^CD24^−/low^ (CSCs) and CD44^−/+^CD24^high^ (non-CSCs) cells were sorted and cultured again in normoxic or hypoxic conditions for 3 days. The culture of non-CSCs in hypoxic conditions led to the dedifferentiation of a considerable percentage of non-CSC cells into CD44^+^CD24^−/low^ CSCs, while the non-CSC population remained very high in normoxia (Figure 4C, 4D). On the other hand, CD44^+^CD24^−/low^ CSCs cultured in normoxic conditions were able to produce both CD44^+^CD24^−/low^ cells and more differentiated non-CSC cells that expressed high levels of CD24. In contrast, when CD44^+^CD24^−/low^ cells were kept in hypoxic conditions, a significantly high percentage of cells maintained their CSC phenotype (Figure 4C, 4D). To ascertain whether hypoxic conditions can also lead to the dedifferentiation of other cell subpopulations, ALDH^+^ and ALDH^−^ T47D cells were sorted and cultured under normoxic or hypoxic conditions. Consistent with the results observed with CD44^+^CD24^−/low^ cells, hypoxia increased the proportion of ALDH^+^ CSCs in a cell population that was originally ALDH^−^ (non-CSCs). In addition, differentiation of sorted ALDH^+^ CSCs into ALDH^−^ cells was lower in hypoxic than in normoxic conditions (Figure 4E, 4F). Among the 19 ALDH isoforms expressed in humans, ALDH1A3 has been shown to correlate best with ALDH activity of patient breast tumor CSCs and cell lines [31]. We therefore examined ALDH1A3 expression in the sorted ALDH^+^ and ALDH^−^ T47D cell subpopulations, which were subsequently cultured under normoxic or hypoxic conditions. Quantitative PCR analyses showed increased ALDH1A3 levels under hypoxia in all cases, even in non-CSC, and furthermore, ALDH1A3 expression levels correlated with ALDH activity (Supplementary Figure 4C), supporting the notion that ALDH activity is primarily due to isoform ALDH1A3 [31]. These findings show that hypoxia expands the pool of CSCs by limiting their differentiation and promoting dedifferentiation of breast cancer cells.

### Hypoxia expands the pool of CSCs both through HIF-dependent and independent mechanisms

**Figure 5:**
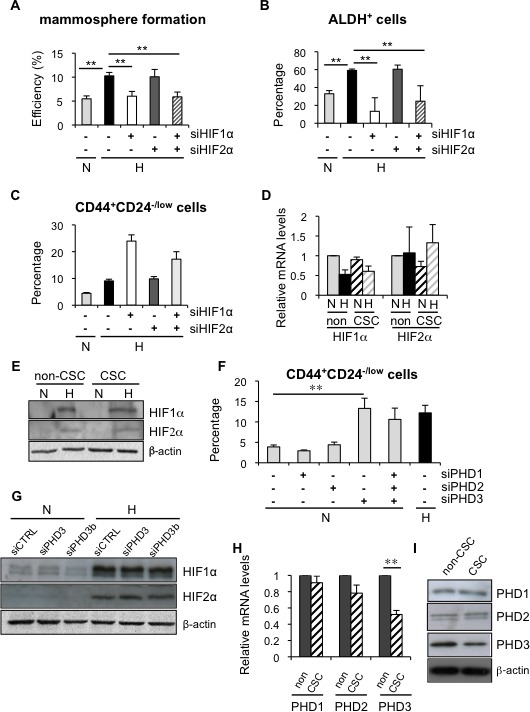
Hypoxia induced dedifferentiation employs both HIF-dependent and independent mechanisms **A.** Mammosphere formation efficiency in MCF-7 cells transfected with siHIF1α and/or siHIF2α, and cultured in normoxia or hypoxia for 3 days. **B.** Percentage of ALDH^+^ cells in T47D cells transfected with siHIF1α and/or siHIF2α, and cultured in normoxia or hypoxia. **C.** Percentage of CD44^+^CD24^−/low^ cells from MDA-MB-468 cells transfected with siHIF1α and/or siHIF2α, and cultured in normoxia or hypoxia. Data are presented as mean ±SEM of 8 independent experiments. (D, E) HIF1α and HIF2α mRNA **D.** and protein **E.** expression in CSCs and non-CSCs from MDA-MB-468 cells cultured in normoxia or hypoxia. **F.** Percentage of CD44^+^CD24^−/low^ MDA-MB-468 cells grown in normoxia after silencing all three PHDs individually or collectively or in hypoxia. **G.** Protein expression of HIF1α and HIF2α in MDA-MB-468 cells transfected with a control siRNA or a siRNA directed to PHD3 and cultured in normoxia or hypoxia. **H.**, **I.** PHD1, PHD2 and PHD3 mRNA **H.** and protein **I.** expression levels in CSCs and non-CSCs sorted from MDA-MB-468 cells. B, D, F and H show means ±SD of three independent experiments.

HIF factors are key mediators of most adaptive changes that occur in response to hypoxia and, indeed, we show induction of HIF1α (Figure 3 and Supplementary Figure 3). Therefore, to determine whether HIF1α and/or HIF2α were implicated in the observed alterations in CSC content under hypoxic conditions, we silenced their expression using specific siRNA sequences. Efficient downregulation of HIF1α (Supplementary Figure 5A, 5C) abrogated the hypoxia-dependent increase in mammosphere formation (Figure 5A) and the percentage of ALDH^+^ cells (Figure 5B), while silencing of HIF2α (Supplementary Figure 5B, 5C) did not affect either of these two subpopulations (Figure 5A, 5B). Surprisingly, silencing of HIF1α and/or HIF2α did not abrogate the increase in CD44^+^CD24^−/low^ CSCs observed in MDA-MB-468 cells under hypoxic conditions (Figure 5C). Furthermore, CD44^+^CD24^−/low^ CSCs and non-CSCs expressed similar levels of HIF1α and HIF2α, both at RNA (Figure 5D) and protein (Figure 5E) levels. Taken together, these observations suggest that the increase in mammosphere formation and ALDH^+^ cell content under hypoxic conditions depends on hypoxia-mediated stabilization of HIF1α. In contrast, the finding that HIF silencing did not affect CD44^+^CD24^−/low^ cell content suggests that some HIF-independent activity is implicated in the regulation of this subpopulation.

Regulation of HIF proteins is the best-known function of prolyl-4-hydroxylases PHD1, PHD2 and PHD3; nevertheless, during the last few years some HIF-independent functions have been described for these proteins. To analyze the potential contribution of the PHDs, all three *PHD* genes were silenced (Supplementary Figure 5D-5F) and alterations in CD44^+^CD24^−/low^ cell content were evaluated by FACS. While *PHD1* and *PHD2* silencing did not exert any significant effect on CSC content, *PHD3* silencing led to a significant increase in the percentage of CD44^+^CD24^−/low^ CSCs in normal oxygen conditions, comparable to that observed under hypoxic conditions (Figure 5F). The same result was obtained when the analysis was repeated using an independent PHD3-specific siRNA sequence (Supplementary Figure 5G). Crucially, silencing of *PHD3* did not result in increased HIF1α or HIF2α expression (Figure 5G), confirming that hypoxia-dependent expansion of CD44^+^CD24^−/low^ CSCs was HIF-independent. Furthermore, PHD3 silencing also increased the CD44^+^CD24^−/low^ population in BT549 cells, confirming that the effect of PHD3 on CSCs is not cell type specific (Supplementary Figure 5H). Analysis of PHD3 expression in CD44^+^CD24^−/low^ CSCs and non-CSCs by qPCR (Figure 5H) and western blot (Figure 5I) indicated that PHD3 expression levels were lower in CSCs than in non-CSCs, while expression of PHD1 and PHD2 did not significantly differ between the two populations (Figure 5H, 5I). Interestingly, PHD3 silencing did not influence the proportion of the CD44^+^CD24^−/low^ cell population in ER-positive MCF7 cells (Supplementary Figure 5I). In conclusion, downregulation of PHD3 increases the proportion of CSCs in ER-negative cells, suggesting that hypoxia can influence the CSC content in a HIF-independent manner through changes in PHD3 expression levels.

### PHD3 silencing mimics hypoxia-driven expansion of CSCs by reducing CD24 expression

**Figure 6:**
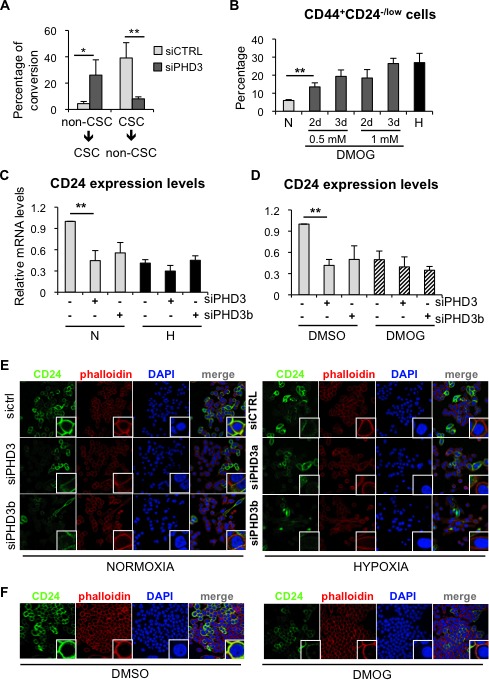
PHD3 silencing promotes dedifferentiation in breast cancer cells through a hydroxylase-dependent mechanism **A.** Graph representing the capacity of CD44^+^CD24^−/low^ CSCs to produce CD44^+^CD24^high^ non-CSCs, and vice-versa, in MDA-MB-468 cells transfected with a control siRNA or a siRNA directed to PHD3. **B.** Percentage of CD44^+^CD24^−/low^ CSCs in MDA-MB-468 cells treated with DMOG. **C.** CD24 mRNA expression levels in MDA-MB-468 cells transfected with two specific siRNA sequences against PHD3 and cultured in normoxia or hypoxia. **D.** CD24 mRNA expression levels in MDA-MB-468 cells transfected with two specific siRNA sequences against PHD3 and treated with the carrier DMSO or 1mM DMOG. **E.** immunofluorescence analysis of CD24 expression in MDA-MB-468 cells cultured in normoxia or hypoxia after PHD3 silencing. **F.** CD24 immunostaining in MDA-MB-468 cells treated with DMSO or DMOG. A, B, C, D, show means ±SD of at least 3 independent experiments, *P < 0.05, **P < 0.01.

Next, we wished to determine the mechanism by which PHD3 influences the CSC content in breast cancer cells. PHD3 silencing promoted the dedifferentiation of non-CSCs to CD44^+^CD24^−/low^ CSCs and prevented the differentiation of the CSCs to non-CSCs (CD44^+^CD24^high^) in MDA-MB-468 cells (Figure 6A), mimicking the effects observed under hypoxic conditions. In order to examine whether the effect of PHD3 on the pool of CSCs depended on its hydroxylase activity, the proportion of CD44^+^CD24^−/low^ cells was measured after treating MDA-MB-468 cells with the pan-hydroxylase inhibitor dimethyloxalylglycine (DMOG), which leads to stabilization of HIF1α (Supplementary Figure 6A). Treatment with DMOG increased the proportion of CD44^+^CD24^−/low^ cells in a dose- and time-dependent manner (Figure 6B), resembling the effect caused by hypoxic conditions. These findings suggest that hypoxia-dependent induction of CD44^+^CD24^−/low^ CSC population implies inhibition of PHD3 hydroxylation.

FACS analysis showed that hypoxia, PHD3 inactivation and DMOG treatment increased the proportion of CD44^+^CD24^−/low^ CSCs by reducing the expression of CD24 at the cell surface, while CD44 expression remained unaltered. To gain insight into the effects of hypoxia signaling on CD24, its expression was analyzed by qPCR and immunofluorescence. Hypoxia, PHD3 downregulation and inhibition of the prolyl-hydroxylase activity by DMOG treatment reduced CD24 expression at the RNA level (Figure 6C, 6D) and protein level (Figure 6E, 6F). However, the combination of PHD3 silencing with hypoxic conditions or DMOG treatment did not further affect CD24 expression when compared to each treatment alone (Figure 6C-6E), suggesting that silencing of PHD3, DMOG and hypoxia are working through the same pathway. In conclusion, hypoxia increases the CD44^+^CD24^−/low^ CSC fraction by reducing CD24 expression levels through the modulation of PHD3 hydroxylase activity.

### Hypoxia and PHD3 silencing regulate CD24 expression through activation of NFκB signaling

**Figure 7:**
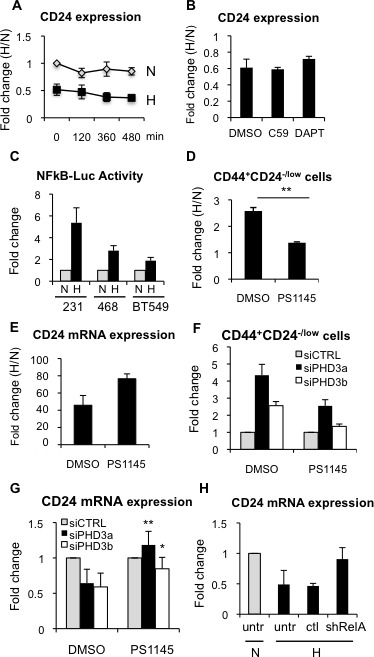
Hypoxia-dependent CD24 downregulation is mediated through NFκB **A.** Analysis of CD24 mRNA stability by qPCR in the presence of actinomycin D during 16 h in normoxic or hypoxic conditions. **B.** CD24 mRNA expression levels in the absence or presence of C59 or DAPT, shown as fold change between hypoxia and normoxia. **C.** NFκB transcriptional activity in MDA-MB-231, MDA-MB-468 and BT549 cells transfected with a NFκB-dependent luciferase reporter. Data are represented as fold change activity in hypoxia versus normoxia. **D.** Percentage of CD44^+^CD24^−/low^ in MDA-MB-468 cells, in the presence or absence of PS1145 (2 μM). Data are presented as fold change between hypoxia and normoxia. **E.** CD24 mRNA expression levels in the absence or presence of PS1145. **F.** Percentage of CD44^+^CD24^−/low^ in MDA-MB-468 cells transfected with specific siRNA sequences against PHD3 in the presence or absence of PS1145. **G.** CD24 mRNA expression in MDA-MB-468 cells transfected with specific siRNA sequences against PHD3 in the presence or absence of PS1145. **H.** CD24 mRNA expression in BT549 cells transfected or not (untr) with a sh control (ctl) or shRelA/p65.

First, we observed that culturing MDA-MB-468 cells in the presence of actinomycin D reduced *CD24* gene expression, independently of oxygen conditions and CD24 mRNA stability was not differentially affected by normoxic or hypoxic conditions (Figure 7A), suggesting that hypoxia reduces CD24 transcription. We next sought to characterize the mechanism involved in the regulation of CD24 expression in response to hypoxia and reduced PHD3 activity. To this end, we investigated different signaling pathways that have been implicated in the regulation of CSCs. FACS analysis showed that the use of specific inhibitors of Wnt (C59) (Supplementary Figure 7A) or Notch (DAPT) (Supplementary Figure 7B) signaling did not abrogate the hypoxia-induced increase in CD44^+^CD24^−/low^ CSCs (Supplementary Figure 7C) or the reduction of CD24 expression levels (Figure 7B). Hypoxia and PHD3 have been shown to regulate NFκB signaling in various tissues. Therefore, we examined NFκB transcriptional activity, which was activated by TNFα (Supplementary Figure 7D) and repressed by the small-molecule IKK inhibitor PS1145 (Supplementary Figure 7E), as expected. We confirmed that NFκB transcriptional activity increased in different breast cancer cell lines cultured under hypoxic conditions (Figure 7C). Inhibition of NFκB signaling by PS1145 (Supplementary Figure 7F, 7G) promoted a statistically significant reduction in the hypoxia-driven expansion of CD44^+^CD24^−/low^ CSCs (Figure 7D), which was due to increased CD24 expression (Figure 7E). In addition, silencing of PHD3, using two different siRNA sequences, also resulted in increased NFκB transcriptional activity (Supplementary Figure 7H, 7I), which could be inhibited by PS1145, leading to reduced CD44^+^CD24^−/low^ cell content (Figure 7F) by releasing the repression of CD24 expression levels (Figure 7G). To evaluate the implication of NFκB signaling using a different strategy, shRelA/p65, which reduces endogenous RelA/p65 levels and therefore target gene expression (Supplementary Figure 7J), was also tested. Analysis of cells transfected with shRelA/p65 showed that lack of functional NFκB activation impaired hypoxia-dependent repression of CD24 expression (Figure 7H). Furthermore, activation of NFkB with TNFα or inhibition by shRelA/p65, was sufficient to reduce or increase CD24 mRNA expression, respectively, even in normal oxygen conditions (Supplementary Figure 7K). Taken together, these results suggest that silencing of PHD3, similar to hypoxic conditions, increase CSC content by reducing expression of CD24 through the activation of NFκB signaling.

## DISCUSSION

In this report we show that hypoxic conditions increase the pool of stem cells both in normal primary epithelial cells and breast cancer cells. The hypoxia-driven increase of CSC populations is a result of limited CSC differentiation and dedifferentiation of breast cancer cells. Hypoxia increases mammosphere formation capacity and the proportion of ALDH^+^ cells through the stabilization of HIF1α, while reducing ER expression and transcriptional activity in ER-positive cells. In contrast, in ER-negative cells, the enrichment in CD44^+^CD24^−/low^ cells by hypoxia involves reduction of the hydroxylase activity of PHD3 and CD24 expression through activation of NFκB signaling.

Hypoxic microenvironments have been shown to influence the behavior of both normal and cancer stem cells in several tissues [32, 33]. We demonstrate that hypoxic conditions increase normal mammary stem/progenitor cell content, leading to the enhanced capacity of primary mammary epithelial cells to form colonies in 3D Matrigel cultures. Hypoxia signaling pathway plays a role during mammary gland development and lactation [34]. In fact, it has been shown that deletion of *Hif1a* from the murine mammary epithelium led to defects in mammary gland development and physiology [35]. In addition, increased expression of genes identified in a hypoxia signature has been correlated with poor prognosis in several types of cancer, including breast cancer [36, 37]. In primary mammary carcinomas hypoxia increases the proportion of CD44^+^CD24^−/low^ESA^+^ CSCs in tumors that have low levels or no active ER signaling [8, 9], according to their ER and PR expression levels detected by immunohistochemistry. However, given the known heterogeneity of breast cancer [5], a more extensive study would be needed to be able to conclude the consequences of hypoxic conditions in different subtypes of breast carcinomas. It has previously been shown that high levels of HIF proteins are implicated in triple negative breast cancer invasiveness and metastasis [38] and that HIF-1α overexpression is observed more often in ER and PR negative carcinomas [39, 40], consistent with our findings that hypoxia reduces ER expression and activity. In fact, the capacity of ER to limit hypoxia-induced expansion of CD44^+^CD24^−/low^ CSCs was further confirmed by depleting ER with the antagonist fulvestrant. Several groups have demonstrated increased breast CSC content in response to hypoxia, but the populations of CSCs analyzed differed. Thus, hypoxia-induced expansion of ALDH^+^ cells was observed both in ER-positive and ER-negative cells [27, 28], and increases in the percentage of CD44^+^CD24^−^ cells and mammosphere formation capacity were shown in ER-negative cells [27, 41, 42], in agreement with our results and in contrast to one report, which detected a decrease in ALDH^+^ cells and mammosphere formation in ER-negative cancers [30]. We and others have shown that HIF1α stabilization mediates the hypoxia-dependent effects in mammospheres and ALDH^+^ cells [27, 28, 30]. Using ER-negative cells derived from late stage tumors spontaneously formed in MMTV-PyMT mice, deletion of HIF1α has been shown to reduce tumor growth in allotransplantation experiments, while decreasing mammosphere formation *in vitro* [43]. Furthermore, it was demonstrated that the increase in ALDH^+^ cells under hypoxic conditions is, at least partly regulated by the Akt/β-catenin signaling pathway in a HIF1α-dependent manner [27], providing a potential mechanism for hypoxia-induced increase in this CSC population. These findings support a correlation between HIF1α expression levels, ALDH^+^ cells, mammosphere formation capacity and tumorigenicity.

ER expression is considered characteristic of well-differentiated luminal tumors and both normal and breast cancer stem and progenitor cells do not express or express low levels of ER [9, 10, 13, 44, 45]. It has been shown that under hypoxic conditions, ER expression is reduced due to proteasomal degradation, leading to reduced ER transcriptional activity [46-48], although other reports argue that the decrease in ER expression is coupled to an increase in its activity [30, 49]. Our data shows that hypoxia reduces ER expression and transcriptional activity. Using different ER-positive breast cancer cell lines, we demonstrate that hypoxia downregulates ER expression, at least in part, at the RNA level, in agreement with previous data [50], resulting in decreased ER transcriptional activity. These results indicate that hypoxia reduces ER expression and activity, which explains the significant correlation observed between HIF-1α immunoreactivity and the absence of ER and PR [40]. Furthermore, it has been observed that well vascularized intratumoral regions contain larger number of ER-positive cells than areas with low blood flow and necrosis [51].

An important question was how the CSC fraction is altered by hypoxia. Changes in the CSC content due to hypoxic conditions are not related to significant alterations in proliferation or apoptosis, but are rather due to the dedifferentiation of cancer cells. Importantly, hypoxic conditions also prevent the differentiation of sorted CSCs. The observation that hypoxia affects the differentiation status of the cells highlights the plasticity of CSCs. Indeed, recently it has been shown that breast CSCs display a cellular plasticity that enables them to transition between epithelial-like and mesenchymal-like states regulated by the tumor microenvironment [52]. In addition, CSC plasticity has recently been demonstrated by elegant intravital lineage tracing experiments in unperturbed mammary tumors [53]. Furthermore, the capacity of hypoxia to drive a reversible phenotype that increases stem-like properties of cells to favor tumor survival has also been observed in other tumors, such as neuroblastoma [54, 55].

Interestingly, we found that silencing PHD3 mimics hypoxia, preventing differentiation of CSCs and leading to dedifferentiation of breast cancer cells. We present evidence that neither HIF1α nor HIF2α are involved in the hypoxia-induced expansion of CD44^+^CD24^−/low^ cells in ER-negative breast cancer cells. This finding was surprising, considering the central role of HIF transcription factors in the regulation of normal and cancer stem cells from several tissues [33, 56-58]. Yet, this result does not necessarily imply that HIFs are not involved in hypoxia-induced increase in stem cell content in ER-negative breast tumors. In fact, we and others have shown that hypoxia increases the percentage of different population of CSCs, ALDH^+^ cells, as well as cells with enhanced mammosphere formation capacity, through HIF1α stabilization, both in ER-positive and ER- negative breast cancer cells [27]. However, in ER-negative breast cancer cells, hypoxia can enlarge different subpopulations of CSCs through two different mechanisms. These findings suggest that therapeutic approaches directed to HIF1α inactivation would be insufficient to prevent the expansion of CD44^+^CD24^−/low^ CSCs. It has been argued that this hypoxia-dependent expansion in CSCs could at least in part explain why patients treated with antiangiogenic factors relapse and present tumors that are more aggressive than the original tumor [27, 59, 60].

The maintenance of CD44^+^CD24^−/low^ cells implicates PHD3, while it is independent of HIF. Interestingly, PHD3 expression has been found to correlate with lower tumor grade and ER positivity [24], and PHD3 transcription is activated by ER both *in vitro* and *in vivo* [61]. These findings are consistent with our observations that low PHD3 levels are found in ER-negative CD44^+^CD24^−/low^ cells and that PHD3 silencing results in dedifferentiation of breast cancer cells. In pancreatic tumors it has been observed that undifferentiated tumors express lower PHD3 levels than well-differentiated tumors, while silencing of PHD3 expression accelerated cell growth, independently of HIF-1 activation [62]. Inhibition of NFκB signaling prevented hypoxia-driven enrichment of CD44^+^CD24^−/low^ cells and reduction of CD24 expression. We propose that down-regulation of PHD3 leads to activation of NFκB, in a HIF-independent manner, which results in the expansion of CD44^+^CD24^−/low^ cells in ER-negative cells (Supplementary Figure 8). A similar effect has been reported in skeletal muscle, where PHD3 was found to promote myoblast differentiation by downregulating NFκB activity [63]. Importantly, increased NFκB activity has been associated with expansion of CSCs in several types of tumors, including breast cancer [64-67]. Tumor-initiating cells are characterized by low levels of membrane CD24 expression [14, 15]. The observed expansion of CD44^+^CD24^−/low^ cells appears to be due to the reduction of membrane CD24 expression through the activation of NFκB signaling. Indeed, activation or inhibition of NFkB signaling reduces or increases CD24 expression, respectively, even in normoxic conditions, supporting a role for NFkB in the regulation of CD24. Interestingly, it has also been shown that CD24 expression can attenuate cell viability and NFkB signaling, but only in CD44-expressing cells [68], thus suggesting a regulatory loop between CD24 and NFkB that may be particularly relevant in CSCs.

A decade ago, the isolation of tumorigenic breast cancer cells with the phenotype CD44^+^CD24^−/low^ represented a first step towards the characterization of breast CSCs [14], which was complemented soon after with the discovery of their capacity to grow as mammospheres [69] and their enhanced aldehyde dehydrogenase activity [13]. Importantly, all these subpopulations of breast CSCs were more efficient at initiating tumors in NOD/SCID mice than non-CSC populations. Nevertheless, genetic profiling has shown that CD44^+^CD24^−/low^ and ALDH^+^ cells represent distinct breast CSCs that, furthermore, are not static, but instead display a cellular plasticity allowing them to transit between epithelial and mesenchymal states [52] and to respond differentially to γ-secretase inhibitors [70]. These findings suggest that the complexity of breast CSCs is higher than initially anticipated [71, 72]. Furthermore, this also implies that these distinct CSC subpopulations, which are using specific molecular pathways, are likely to present distinct anti-cancer drug responsiveness. This work also has potential clinical implications given that it has been proposed that treatment with antiangiogenic agents should be combined with CSC-targeting drugs, since HIF1α increases breast CSCs [27], and that chemotherapy should be combined with HIF inhibitors in women with triple negative breast cancer [28]. Therefore, we propose that the design of combinatorial therapies targeting CSCs should take into account their intrinsic heterogeneity in order to achieve the wide spectrum required to avoid or limit CSC expansion in the tumor and development of resistance to therapy leading to metastasis.

## MATERIALS AND METHODS

### Isolation of human breast epithelial cells and Ethics Statement

Normal breast tissue was obtained from women (24 - 43 years old) undergoing reduction mammoplasty with no previous history of breast cancer (clinical data can be found in Supplementary Table 1). Tumor samples were obtained from core biopsies or from women who underwent therapeutic surgery (histopathological information can be found in Supplementary Table 2). Investigation has been conducted in accordance with the ethical standards and according to the Declaration of Helsinki and according to national and international guidelines. All patients provided written informed consent and the procedures were approved by the local Hospital Research Ethics Committee, by the “Ethics Committee of Clinical Investigations of Euskadi” and by the Centre´s review board. Upon arrival, all samples were immediately processed as previously described [44].

### Adherent cell culture

MCF-7, T47D, MDA-MB-468, MDA-MB-231, ZR75-1 and SK-BR-3 cell lines were obtained from American Type Culture Collection (ATCC) and cultured in DMEM/F-12 medium with GlutaMAX (Gibco) supplemented with 8% FBS (Sigma) and 1% penicillin/streptomycin (Gibco) at 37°C in 5% CO_2_. BT549 cells (kindly provided by A Carracedo) were cultured in DMEM/F-12 medium with GlutaMAX supplemented with 10% FBS and 1% penicillin/streptomycin. For experiments with hormonal treatments, MCF-7 and T47D cells were hormone-depleted for 2-3 days in phenol-red free (PRF) DMEM/F-12 medium (Gibco) supplemented with 8% charcoal (Sigma) stripped FBS, before adding 17-β-estradiol (estrogen) (Sigma), or fulvestrant (ICI 182,780, kindly provided by AE Wakeling). Primary normal epithelial cells were cultured in DMEM/F-12 with GlutaMax supplemented with 5% FBS, 5 μg/ml insulin (Sigma), 1 μg/ml hydrocortisone (Sigma), 10 ng/ml EGF (Invitrogen), 100 ng/ml cholera toxin (Sigma) and 1% penicillin/streptomycin.

Cells were treated with 2 μM PS1145, 100 nM C59 [80] or 10 μM DAPT (Sigma). Media were changed every 24 h - 36 h to ensure the activity of all compounds. C59 was kindly provided by R Kypta and the other drugs are from Sigma.

### Mammosphere culture

Cells were detached with TrypLE™ Select (Invitrogen) and plated in 75 cm^2^ in flasks treated with poly(2-hydroxyethylmethacrylate) (poly-HEMA [Sigma]) at a density of 3,000 cells/ml. Alternatively, 500 cells were sorted into poly-HEMA coated 6-well plates. MCF-7 and MDA-MB-468 cells were grown in DMEM/F-12 medium with GlutaMAX, supplemented with B27 (Gibco), 10 ng/ml EGF (Invitrogen), 2 ng/ml bFGF (Millipore) at 37°C in 5% CO_2_. When culturing primary cells, medium was enriched with B27, 20 ng/ml EGF, 20 ng/ml bFGF. After 5 to 8 days, mammospheres were stained with crystal violet solution, immobilized in 0,3% agar, and counted.

### Hypoxia treatment

Hypoxic cultures were carried out in a humidified hypoxia workstation (*In Vivo* 400, Ruskin) with an atmosphere of 1% oxygen and 5% CO_2_ balanced with nitrogen. When used, and unless otherwise stated, cells were treated with the hypoxia mimetic dimethyloxaloylglycine (DMOG) at 1 mM for 3 days, replacing the media every 24h.

### Colony formation assay with normal breast epithelial cells

Following cell culture in adherence or suspension conditions for 3-4 days, as described above, dissociated single cells were plated in Millicell EZ SLIDE chamber slides (Millipore) (1000 cells per well) on top of a layer of growth factor reduced Matrigel (BD) that was previously allowed to polymerize. Cells were cultured for 10-12 days in different oxygen conditions in medium supplemented with 5% FBS, 5 μg/ml insulin (Sigma), 1 μg/ml hydrocortisone (Sigma), 10 ng/ml EGF (Invitrogen), 100 ng/ml cholera toxin (Sigma), 5% Matrigel and 1% penicillin/streptomycin.

### RNA extraction and qPCR

Total RNA was isolated using TRIzol (Invitrogen). When the cell number was low, RNeasy Micro Kit (Qiagen) was used. In all cases, RNA extraction was performed according to the instructions of the manufacturer. DNAse-treated RNA was used to synthesize cDNA using M-MLV reverse transcriptase (Invitrogen) or Omniscript reverse transcriptase, following the manufacturer's protocol. qPCR was performed on a 7300 Real-Time PCR System (Applied Biosystems) or a ViiA 7™ Real-Time PCR System (Applied Biosystems), using iTaq™ SYBR^®^ Green Supermix with ROX (BioRad) or PerfeCta^®^ SYBR^®^ Green SuperMix with Low Rox (Quanta Biosciences), respectively. Primer sequences used for PCR amplification are summarized in Supplementary Table 3.

### Transcription assays

All cells were seeded at 40.000 cells per well in 24-well tissue-culture plates, hormone-depleted for 2-3 days and transfected using Genejuice reagent (Merck), following the indications of the manufacturer. Cells were transfected with the pGL2-ERE TK-luciferase vector containing the thymidine kinase (TK) promoter and three copies of a consensus ERE (Estrogen Responsive Element), driving the expression of the luciferase gene (kindly provided by M Parker). A vector expressing beta-galactosidase was used as control for transfection efficiency [73]. After transfection cells were treated with or without estrogen and cultured in normoxic or hypoxic conditions for 40 h, after which luciferase and beta-galactosidase activities were measured using the Luciferase Assay Kit (Promega) and the Tropix Galacto-Light-Plus Assay (Applied Biosystems), respectively. Luminescence was measured in a Veritas TM Microplate Luminometer (Turner Biosystems).

MDA-MB-468, MDA-MB-231 and BT-549 cells were seeded in 6-well plate at 3×10^5^ cells/well and were transfected with the NFkB-TK-luciferase reporter [74] (kindly provided by R Kypta) using Lipofectamine LTX (Invitrogen) following the manufacturer's instructions. Each well also received *renilla* to normalize for transfection efficiency. After transfection, the cells were maintained in DMEM:F12 containing 8% FBS for 48 h. The cell lysates were assayed for luciferase and renilla activities with a luciferase reporter assay kit (Dual-Luciferase Reporter Assay System; Promega) using a luminometer (Turner Biosystem).

To assay for NFkB activity BT-549 cells were seeded in 6-well plates at 3×10^5^ cells/well and were transfected with the *shRelA* (p65 shRNA) *plasmid [75],* kindly provided by B Lewis) using Lipofectamine LTX (Invitrogen) following the manufacturer's instructions. After 5 h, cells were maintained in normoxic or hypoxic conditions for 40 h. GFP positive (transfected) or negative (not transfected) cells were sorted using a FACS ARIA and collected to perform total RNA extraction.

### Gene silencing

siRNA transfection was performed using Lipofectamine 2000 or Lipofectamine RNAimax (Invitrogen), following the guidelines of the manufacturer for reverse transfection. For the mammosphere formation assays after gene silencing, cells were transfected twice, to ensure efficient silencing during the whole experiment. Following incubations with liposomes, cells were collected by centrifugation (400 x g for 5 minutes). 3000 cells/ml were seeded in 75 cm^2^ poly-HEMA coated flasks in order to allow mammosphere formation. The RNAi sequences used in this study are shown in Supplementary Table 4.

### Western blotting

Cell lysates were prepared with Laemmli buffer (50 mM tris pH 6,8, 1,25% SDS, 15% glycerol). Protein extracts of cells cultured in hypoxia were made inside the hypoxic chamber to avoid reoxygenation. All extracts were heated at 95°C for 15 minutes for a complete lysis and denaturalization and Lowry protein assay (BioRad) was used for the quantification of protein extracts, followed by addition of β- mercaptoethanol (Applichem) (5% final concentration) and bromophenol blue (Sigma). Blots were then incubated with the following primary antibodies: ERα (Novocastra, clone 6F11), HIF1α (antiserum 2087 [76]), HIF2α (kindly provided by D Richard), PHD1 (Bethyl, A300-326A), PHD2 (antiserum 804 [77]), PHD3 (Novus Biologicals, NB-100-139), PR (Novocastra, clone 16), RARα (Santa Cruz, sc-6551), β-actin (AC-15/A5441), and β-tubulin (Sigma). Proteins were detected using ECL (Amersham) and visualized on X-ray film or by acquiring digital images with the Molecular Imager ChemiDoc XRS System.

### Immunofluorescence

For immunofluorescence experiments, cells were processed as previously described [78]. Briefly, cells grown on cover slides were fixed in 4% paraformaldehyde (Santa Cruz), permeabilised with PBS 0,5% saponin and blocked in blocking buffer (PBS containing 2%BSA, 50 mM Glycine and 0,1% saponin). Cells were then incubated with anti-CD24 antibody (Dianova, T-1358) overnight at 4°C. After washing with PBS-0,1% saponin, cells were incubated with anti-mouse Alexa 488 (Molecular Probes, A21202) and phalloidin (phalloidin-Tetramethylrhodamine B isothiocyanate, Sigma, P1951). Slides were washed and finally mounted in Vectashield with DAPI (Vector) and visualized on a Leica confocal microscope.

### Fluorescence activated cell sorting (FACS)

For CD44/CD24/ESA, CD44/CD24 and CD49f/ESA stainings, PE-conjugated anti-CD24 antibody (BD, 555428), APC-conjugated anti-CD44 antibody (BD, 559942), FITC-conjugated anti-ESA antibody (Biomeda Corp, FM010) and APC-conjugated anti-CD49f antibody (eBioscience, 17-0495-80) were used [79]. EMA/CALLA labeling was performed as previously described [10], using a rat monoclonal antibody against EMA (ICR2 [10]), followed by FITC-conjugated anti-rat antibody (Southern Biotech, 3010-02), and PE-conjugated anti-CALLA antibody (DAKO, R0848). In all cases, control samples were stained with isotype-matched control antibodies, the viability dye 7-aminoactinomycin D (7AAD) (BD) was used for dead cell exclusion, and fluorescence minus one (FMO) controls were used to define the gates [79].

For BrdU incorporation assay, cells were treated with 10 μM bromodeoxyuridine (BrdU) (Amersham) for 1 h. Upon harvesting, cells were fixed in 4% PFA for 15 minutes, permeabilized in 0,1% triton for 20 minutes, and incubated with Fastimmun™ anti-BrdU-FITC antibody with DNAse (BD, 340649) for 30 minutes. Cells not treated with BrdU were used as negative control. Annexin V staining was performed using FITC Annexin V Apoptosis Detection kit (BD, 556547), following the instructions of the manufacturer and using 7AAD instead of Propidium Iodide.

To measure ALDH activity in cells, ALDEFLUOR assay (Stemcell Technologies) was carried out according to manufacturer's guidelines, and as previously described [44].

In all cases, cells were analyzed using FACSCanto II (Becton Dickinson) or FACSAria (Becton Dickinson) flow cytometres. FACSAria was used for sorting cells. Data were analyzed using the FACSDiva software.

### Statistical analysis

Data from at least three independent experiments are expressed as means ± SD (or ± SEM, if indicated). Each data point of real-time PCR, mammosphere formation and luciferase activity assays was run at least in triplicate. Student´s *t*-test was used to determine statistically significant differences and *p* < 0.05 was considered to be significant unless otherwise specified.

## SUPPLEMENTARY MATERIAL FIGURES AND TABLES



## Abbreviations

SC: stem cell
CSC: cancer stem cell
ER: estrogen receptor
HIF: hypoxia-inducible factor
PR: progesterone receptor
RAR: retinoic acid receptor
AREG: amphiregulin
pS2, also known as TFF1: trefoil factor 1
DMOG: dimethyloxalylglycine

## References

[1] Ferlay J, Soerjomataram I, Dikshit R, Eser S, Mathers C, Rebelo M, Parkin DM, Forman D, Bray F (2015). Cancer incidence and mortality worldwide: Sources, methods and major patterns in GLOBOCAN 2012. Int J Cancer.

[2] Perou CM, Sorlie T, Eisen MB, van de Rijn M, Jeffrey SS, Rees CA, Pollack JR, Ross DT, Johnsen H, Akslen LA, Fluge O, Pergamenschikov A, Williams C, Zhu SX, Lonning PE, Borresen-Dale AL (2000). Molecular portraits of human breast tumours. Nature.

[3] Sorlie T, Perou CM, Tibshirani R, Aas T, Geisler S, Johnsen H, Hastie T, Eisen MB, van de Rijn M, Jeffrey SS, Thorsen T, Quist H, Matese JC, Brown PO, Botstein D, Lonning PE (2001). Gene expression patterns of breast carcinomas distinguish tumor subclasses with clinical implications. Proc Natl Acad Sci U S A.

[4] Sorlie T, Tibshirani R, Parker J, Hastie T, Marron JS, Nobel A, Deng S, Johnsen H, Pesich R, Geisler S, Demeter J, Perou CM, Lonning PE, Brown PO, Borresen-Dale AL, Botstein D (2003). Repeated observation of breast tumor subtypes in independent gene expression data sets. Proc Natl Acad Sci U S A.

[5] Curtis C, Shah SP, Chin SF, Turashvili G, Rueda OM, Dunning MJ, Speed D, Lynch AG, Samarajiwa S, Yuan Y, Graf S, Ha G, Haffari G, Bashashati A, Russell R, McKinney S (2012). The genomic and transcriptomic architecture of 2,000 breast tumours reveals novel subgroups. Nature.

[6] Ali H, Rueda OM, Chin SF, Curtis C, Dunning MJ, Aparicio S, Caldas C (2014). Genome-driven integrated classification of breast cancer validated in over 7,500 samples. Genome biology.

[7] Payne SJ, Bowen RL, Jones JL, Wells CA (2008). Predictive markers in breast cancer—the present. Histopathology.

[8] Simoes BM, Vivanco MD (2011). Cancer stem cells in the human mammary gland and regulation of their differentiation by estrogen. Future Oncol.

[9] Piva M, Domenici G, Iriondo O, Rabano M, Simoes BM, Comaills V, Barredo I, Lopez-Ruiz JA, Zabalza I, Kypta R, Vivanco MD (2014). Sox2 promotes tamoxifen resistance in breast cancer cells. EMBO Mol Med.

[10] Clayton H, Titley I, Vivanco M (2004). Growth and differentiation of progenitor/stem cells derived from the human mammary gland. Exp Cell Res.

[11] Eirew P, Stingl J, Raouf A, Turashvili G, Aparicio S, Emerman JT, Eaves CJ (2008). A method for quantifying normal human mammary epithelial stem cells with *in vivo* regenerative ability. Nat Med.

[12] Lim E, Vaillant F, Wu D, Forrest NC, Pal B, Hart AH, Asselin-Labat ML, Gyorki DE, Ward T, Partanen A, Feleppa F, Huschtscha LI, Thorne HJ, Fox SB, Yan M, French JD (2009). Aberrant luminal progenitors as the candidate target population for basal tumor development in BRCA1 mutation carriers. Nat Med.

[13] Ginestier C, Hur MH, Charafe-Jauffret E, Monville F, Dutcher J, Brown M, Jacquemier J, Viens P, Kleer CG, Liu S, Schott A, Hayes D, Birnbaum D, Wicha MS, Dontu G (2007). ALDH1 is a marker of normal and malignant human mammary stem cells and a predictor of poor clinical outcome. Cell Stem Cell.

[14] Al-Hajj M, Wicha MS, Benito-Hernandez A, Morrison SJ, Clarke MF (2003). Prospective identification of tumorigenic breast cancer cells. Proc Natl Acad Sci U S A.

[15] Fillmore CM, Kuperwasser C (2008). Human breast cancer cell lines contain stem-like cells that self-renew, give rise to phenotypically diverse progeny and survive chemotherapy. Breast Cancer Res.

[16] Charafe-Jauffret E, Ginestier C, Iovino F, Wicinski J, Cervera N, Finetti P, Hur MH, Diebel ME, Monville F, Dutcher J, Brown M, Viens P, Xerri L, Bertucci F, Stassi G, Dontu G (2009). Breast cancer cell lines contain functional cancer stem cells with metastatic capacity and a distinct molecular signature. Cancer Res.

[17] Semenza GL (2013). Cancer-stromal cell interactions mediated by hypoxia-inducible factors promote angiogenesis, lymphangiogenesis, and metastasis. Oncogene.

[18] Bertout JA, Patel SA, Simon MC (2008). The impact of O2 availability on human cancer. Nature reviews Cancer.

[19] Semenza GL (2013). HIF-1 mediates metabolic responses to intratumoral hypoxia and oncogenic mutations. The Journal of clinical investigation.

[20] Bruick RK, McKnight SL (2001). A conserved family of prolyl-4-hydroxylases that modify HIF. Science.

[21] Huang LE, Gu J, Schau M, Bunn HF (1998). Regulation of hypoxia-inducible factor 1alpha is mediated by an O2-dependent degradation domain via the ubiquitin-proteasome pathway. Proc Natl Acad Sci U S A.

[22] Salceda S, Caro J (1997). Hypoxia-inducible factor 1alpha (HIF-1alpha) protein is rapidly degraded by the ubiquitin-proteasome system under normoxic conditions. Its stabilization by hypoxia depends on redox-induced changes. The Journal of biological chemistry.

[23] Semenza GL (2012). Hypoxia-inducible factors in physiology and medicine. Cell.

[24] Peurala E, Koivunen P, Bloigu R, Haapasaari KM, Jukkola-Vuorinen A (2012). Expressions of individual PHDs associate with good prognostic factors and increased proliferation in breast cancer patients. Breast Cancer Res Treat.

[25] Chan DA, Kawahara TL, Sutphin PD, Chang HY, Chi JT, Giaccia AJ (2009). Tumor vasculature is regulated by PHD2-mediated angiogenesis and bone marrow-derived cell recruitment. Cancer Cell.

[26] Henze AT, Garvalov BK, Seidel S, Cuesta AM, Ritter M, Filatova A, Foss F, Dopeso H, Essmann CL, Maxwell PH, Reifenberger G, Carmeliet P, Acker-Palmer A, Acker T (2014). Loss of PHD3 allows tumours to overcome hypoxic growth inhibition and sustain proliferation through EGFR. Nature communications.

[27] Conley SJ, Gheordunescu E, Kakarala P, Newman B, Korkaya H, Heath AN, Clouthier SG, Wicha MS (2012). Antiangiogenic agents increase breast cancer stem cells via the generation of tumor hypoxia. Proc Natl Acad Sci U S A.

[28] Samanta D, Gilkes DM, Chaturvedi P, Xiang L, Semenza GL (2014). Hypoxia-inducible factors are required for chemotherapy resistance of breast cancer stem cells. Proc Natl Acad Sci U S A.

[29] Vaupel P, Schlenger K, Knoop C, Hockel M (1991). Oxygenation of human tumors: evaluation of tissue oxygen distribution in breast cancers by computerized O2 tension measurements. Cancer Res.

[30] Harrison H, Rogerson L, Gregson HJ, Brennan KR, Clarke RB, Landberg G (2013). Contrasting hypoxic effects on breast cancer stem cell hierarchy is dependent on ER-alpha status. Cancer Res.

[31] Marcato P, Dean CA, Pan D, Araslanova R, Gillis M, Joshi M, Helyer L, Pan L, Leidal A, Gujar S, Giacomantonio CA, Lee PW (2011). Aldehyde dehydrogenase activity of breast cancer stem cells is primarily due to isoform ALDH1A3 and its expression is predictive of metastasis. Stem cells.

[32] Mohyeldin A, Garzon-Muvdi T, Quinones-Hinojosa A (2010). Oxygen in stem cell biology: a critical component of the stem cell niche. Cell Stem Cell.

[33] Li Z, Bao S, Wu Q, Wang H, Eyler C, Sathornsumetee S, Shi Q, Cao Y, Lathia J, McLendon RE, Hjelmeland AB, Rich JN (2009). Hypoxia-inducible factors regulate tumorigenic capacity of glioma stem cells. Cancer Cell.

[34] Shao Y, Zhao FQ (2014). Emerging evidence of the physiological role of hypoxia in mammary development and lactation. Journal of animal science and biotechnology.

[35] Seagroves TN, Hadsell D, McManaman J, Palmer C, Liao D, McNulty W, Welm B, Wagner KU, Neville M, Johnson RS (2003). HIF1alpha is a critical regulator of secretory differentiation and activation, but not vascular expansion, in the mouse mammary gland. Development.

[36] Chi JT, Wang Z, Nuyten DS, Rodriguez EH, Schaner ME, Salim A, Wang Y, Kristensen GB, Helland A, Borresen-Dale AL, Giaccia A, Longaker MT, Hastie T, Yang GP, van de Vijver MJ, Brown PO (2006). Gene expression programs in response to hypoxia: cell type specificity and prognostic significance in human cancers. PLoS medicine.

[37] Buffa FM, Harris AL, West CM, Miller CJ (2010). Large meta-analysis of multiple cancers reveals a common, compact and highly prognostic hypoxia metagene. British journal of cancer.

[38] Montagner M, Enzo E, Forcato M, Zanconato F, Parenti A, Rampazzo E, Basso G, Leo G, Rosato A, Bicciato S, Cordenonsi M, Piccolo S (2012). SHARP1 suppresses breast cancer metastasis by promoting degradation of hypoxia-inducible factors. Nature.

[39] Kaya AO, Gunel N, Benekli M, Akyurek N, Buyukberber S, Tatli H, Coskun U, Yildiz R, Yaman E, Ozturk B (2012). Hypoxia inducible factor-1 alpha and carbonic anhydrase IX overexpression are associated with poor survival in breast cancer patients. Journal of BUON : official journal of the Balkan Union of Oncology.

[40] Trastour C, Benizri E, Ettore F, Ramaioli A, Chamorey E, Pouyssegur J, Berra E (2007). HIF-1alpha and CA IX staining in invasive breast carcinomas: prognosis and treatment outcome. Int J Cancer.

[41] Louie E, Nik S, Chen JS, Schmidt M, Song B, Pacson C, Chen XF, Park S, Ju J, Chen EI (2010). Identification of a stem-like cell population by exposing metastatic breast cancer cell lines to repetitive cycles of hypoxia and reoxygenation. Breast Cancer Res.

[42] Chen X, Iliopoulos D, Zhang Q, Tang Q, Greenblatt MB, Hatziapostolou M, Lim E, Tam WL, Ni M, Chen Y, Mai J, Shen H, Hu DZ, Adoro S, Hu B, Song M (2014). XBP1 promotes triple-negative breast cancer by controlling the HIF1alpha pathway. Nature.

[43] Schwab LP, Peacock DL, Majumdar D, Ingels JF, Jensen LC, Smith KD, Cushing RC, Seagroves TN (2012). Hypoxia-inducible factor 1alpha promotes primary tumor growth and tumor-initiating cell activity in breast cancer. Breast cancer research : BCR.

[44] Simoes BM, Piva M, Iriondo O, Comaills V, Lopez-Ruiz JA, Zabalza I, Mieza JA, Acinas O, Vivanco MD (2011). Effects of estrogen on the proportion of stem cells in the breast. Breast Cancer Res Treat.

[45] Shipitsin M, Campbell LL, Argani P, Weremowicz S, Bloushtain-Qimron N, Yao J, Nikolskaya T, Serebryiskaya T, Beroukhim R, Hu M, Halushka MK, Sukumar S, Parker LM, Anderson KS, Harris LN, Garber JE (2007). Molecular definition of breast tumor heterogeneity. Cancer cell.

[46] Jung YS, Lee SJ, Yoon MH, Ha NC, Park BJ (2012). Estrogen receptor alpha is a novel target of the Von Hippel-Lindau protein and is responsible for the proliferation of VHL-deficient cells under hypoxic conditions. Cell cycle.

[47] Stoner M, Saville B, Wormke M, Dean D, Burghardt R, Safe S (2002). Hypoxia induces proteasome-dependent degradation of estrogen receptor alpha in ZR-75 breast cancer cells. Molecular endocrinology.

[48] Cooper C, Liu GY, Niu YL, Santos S, Murphy LC, Watson PH (2004). Intermittent hypoxia induces proteasome-dependent down-regulation of estrogen receptor alpha in human breast carcinoma. Clinical cancer research : an official journal of the American Association for Cancer Research.

[49] Yi JM, Kwon HY, Cho JY, Lee YJ (2009). Estrogen and hypoxia regulate estrogen receptor alpha in a synergistic manner. Biochemical and biophysical research communications.

[50] Ryu K, Park C, Lee Y (2011). Hypoxia-inducible factor 1 alpha represses the transcription of the estrogen receptor alpha gene in human breast cancer cells. Biochemical and biophysical research communications.

[51] Lloyd MC, Alfarouk KO, Verduzco D, Bui MM, Gillies RJ, Ibrahim ME, Brown JS, Gatenby RA (2014). Vascular measurements correlate with estrogen receptor status. BMC cancer.

[52] Liu S, Cong Y, Wang D, Sun Y, Deng L, Liu Y, Martin-Trevino R, Shang L, McDermott SP, Landis MD, Hong S, Adams A, D'Angelo R, Ginestier C, Charafe-Jauffret E, Clouthier SG (2014). Breast cancer stem cells transition between epithelial and mesenchymal states reflective of their normal counterparts. Stem cell reports.

[53] Zomer A, Ellenbroek SI, Ritsma L, Beerling E, Vrisekoop N, Van Rheenen J (2013). Intravital imaging of cancer stem cell plasticity in mammary tumors. Stem cells.

[54] Jogi A, Ora I, Nilsson H, Lindeheim A, Makino Y, Poellinger L, Axelson H, Pahlman S (2002). Hypoxia alters gene expression in human neuroblastoma cells toward an immature and neural crest-like phenotype. Proc Natl Acad Sci U S A.

[55] Heddleston JM, Li Z, Lathia JD, Bao S, Hjelmeland AB, Rich JN (2010). Hypoxia inducible factors in cancer stem cells. British journal of cancer.

[56] Mazumdar J, Hickey MM, Pant DK, Durham AC, Sweet-Cordero A, Vachani A, Jacks T, Chodosh LA, Kissil JL, Simon MC, Keith B (2010). HIF-2alpha deletion promotes Kras-driven lung tumor development. Proc Natl Acad Sci U S A.

[57] Takubo K, Goda N, Yamada W, Iriuchishima H, Ikeda E, Kubota Y, Shima H, Johnson RS, Hirao A, Suematsu M, Suda T (2010). Regulation of the HIF-1alpha level is essential for hematopoietic stem cells. Cell Stem Cell.

[58] Zhang H, Li H, Xi HS, Li S (2012). HIF1alpha is required for survival maintenance of chronic myeloid leukemia stem cells. Blood.

[59] Casanovas O, Hicklin DJ, Bergers G, Hanahan D (2005). Drug resistance by evasion of antiangiogenic targeting of VEGF signaling in late-stage pancreatic islet tumors. Cancer Cell.

[60] Paez-Ribes M, Allen E, Hudock J, Takeda T, Okuyama H, Vinals F, Inoue M, Bergers G, Hanahan D, Casanovas O (2009). Antiangiogenic therapy elicits malignant progression of tumors to increased local invasion and distant metastasis. Cancer Cell.

[61] Kim M, Neinast MD, Frank AP, Sun K, Park J, Zehr JA, Vishvanath L, Morselli E, Amelotte M, Palmer BF, Gupta RK, Scherer PE, Clegg DJ (2014). ERalpha upregulates Phd3 to ameliorate HIF-1 induced fibrosis and inflammation in adipose tissue. Molecular metabolism.

[62] Su Y, Loos M, Giese N, Hines OJ, Diebold I, Gorlach A, Metzen E, Pastorekova S, Friess H, Buchler P (2010). PHD3 regulates differentiation, tumour growth and angiogenesis in pancreatic cancer. British journal of cancer.

[63] Fu J, Taubman MB (2010). Prolyl hydroxylase EGLN3 regulates skeletal myoblast differentiation through an NF-kappaB-dependent pathway. The Journal of biological chemistry.

[64] Liu M, Sakamaki T, Casimiro MC, Willmarth NE, Quong AA, Ju X, Ojeifo J, Jiao X, Yeow WS, Katiyar S, Shirley LA, Joyce D, Lisanti MP, Albanese C, Pestell RG (2010). The canonical NF-kappaB pathway governs mammary tumorigenesis in transgenic mice and tumor stem cell expansion. Cancer Res.

[65] Yamamoto M, Taguchi Y, Ito-Kureha T, Semba K, Yamaguchi N, Inoue J (2013). NF-kappaB non-cell-autonomously regulates cancer stem cell populations in the basal-like breast cancer subtype. Nature communications.

[66] Kendellen MF, Bradford JW, Lawrence CL, Clark KS, Baldwin AS (2014). Canonical and non-canonical NF-kappaB signaling promotes breast cancer tumor-initiating cells. Oncogene.

[67] Wei L, Liu TT, Wang HH, Hong HM, Yu AL, Feng HP, Chang WW (2011). Hsp27 participates in the maintenance of breast cancer stem cells through regulation of epithelial-mesenchymal transition and nuclear factor-kappaB. Breast Cancer Res.

[68] Ju JH, Jang K, Lee KM, Kim M, Kim J, Yi JY, Noh DY, Shin I (2011). CD24 enhances DNA damage-induced apoptosis by modulating NF-kappaB signaling in CD44-expressing breast cancer cells. Carcinogenesis.

[69] Dontu G, Abdallah WM, Foley JM, Jackson KW, Clarke MF, Kawamura MJ, Wicha MS (2003). *In vitro* propagation and transcriptional profiling of human mammary stem/progenitor cells. Genes Dev.

[70] Azzam DJ, Zhao D, Sun J, Minn AJ, Ranganathan P, Drews-Elger K, Han X, Picon-Ruiz M, Gilbert CA, Wander SA, Capobianco AJ, El-Ashry D, Slingerland JM (2013). Triple negative breast cancer initiating cell subsets differ in functional and molecular characteristics and in gamma-secretase inhibitor drug responses. EMBO Mol Med.

[71] Vivanco M (2010). Function follows form: defining mammary stem cells. Sci Transl Med.

[72] Liu Y, Nenutil R, Appleyard MV, Murray K, Boylan M, Thompson AM, Coates PJ (2014). Lack of correlation of stem cell markers in breast cancer stem cells. British journal of cancer.

[73] Vivanco MD, Johnson R, Galante PE, Hanahan D, Yamamoto KR (1995). A transition in transcriptional activation by the glucocorticoid and retinoic acid receptors at the tumor stage of dermal fibrosarcoma development. Embo J.

[74] Campa VM, Baltziskueta E, Bengoa-Vergniory N, Gorrono-Etxebarria I, Wesolowski R, Waxman J, Kypta RM (2014). A screen for transcription factor targets of glycogen synthase kinase-3 highlights an inverse correlation of NFkappaB and androgen receptor signaling in prostate cancer. Oncotarget.

[75] Ranuncolo SM, Pittaluga S, Evbuomwan MO, Jaffe ES, Lewis BA (2012). Hodgkin lymphoma requires stabilized NIK and constitutive RelB expression for survival. Blood.

[76] Richard DE, Berra E, Gothie E, Roux D, Pouyssegur J (1999). p42/p44 mitogen-activated protein kinases phosphorylate hypoxia-inducible factor 1alpha (HIF-1alpha) and enhance the transcriptional activity of HIF-1. The Journal of biological chemistry.

[77] Berra E, Benizri E, Ginouves A, Volmat V, Roux D, Pouyssegur J (2003). HIF prolyl-hydroxylase 2 is the key oxygen sensor setting low steady-state levels of HIF-1alpha in normoxia. EMBO J.

[78] Elizalde C, Campa VM, Caro M, Schlangen K, Aransay AM, Vivanco M, Kypta RM (2011). Distinct roles for Wnt-4 and Wnt-11 during retinoic acid-induced neuronal differentiation. Stem cells.

[79] Iriondo O, Rabano M, Vivanco MD (2015). FACS Sorting Mammary Stem Cells. Methods in molecular biology.

[80] Bengoa-Vergniory N, Gorroño-Etxebarria I, González-Salazar I, Kypta RM (2014). A switch from canonical to noncanonical Wnt signaling mediates early differentiation of human neural stem cells. Stem Cells.

