# Supplementary material for: Distinct breast cancer stem/progenitor cell populations require either HIF1α or loss of PHD3 to expand under hypoxic conditions
Source: Oncotarget. 2015 Sep 10;6(31):31721–39. doi: 10.18632/oncotarget.5564 (PMC4741635; doi:10.18632/oncotarget.5564)
Supplement: Supplementary file 1 [file oncotarget-06-31721-s001.pdf]

Distinct breast cancer stem/progenitor cell populations require either HIF1a or loss of PHD3 to expand under hypoxic conditions

Supplementary Material

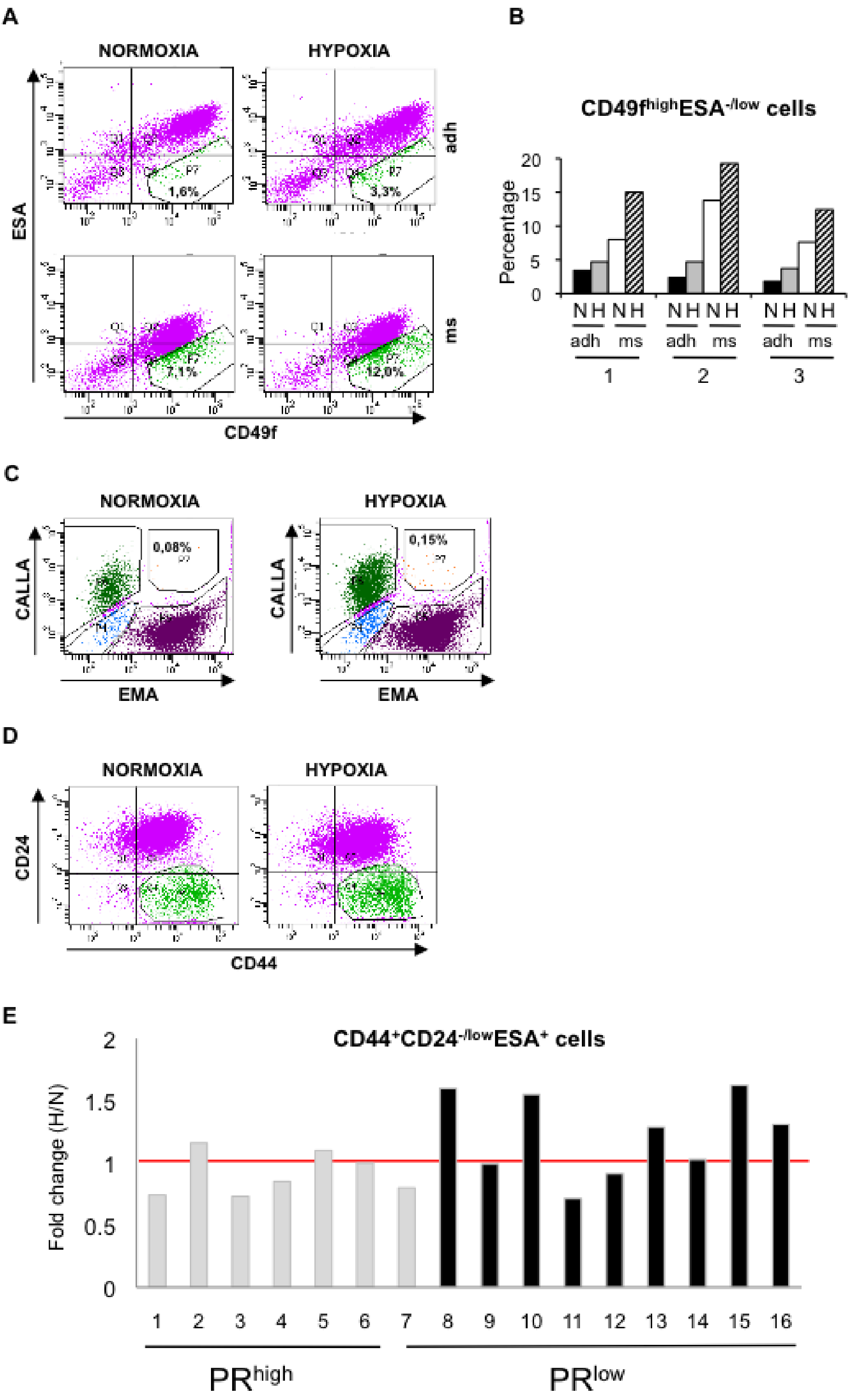

Supplementary Figure 1. Raw data of FACS analyses using primary normal and breast tumor cells. (A) Representative plots of CD49f/ESA stainings performed with normal breast epithelial cells cultured in adherent (adh) or mammosphere (ms) and normoxic or hypoxic conditions. (B) Percentage of $CD49f^{high}ESA^{-/low}$cells in 3 normal breast epithelial cells cultured in different oxygen conditions, in adherent or suspension conditions. (C, D) Representative plots of EMA/CALLA (c) and CD44/CD24 (d) stainings performed with normal breast epithelial cells cultured in normoxic or hypoxic conditions. (E) $CD44^{+}CD24^{-/low}ESA^{+}$ CSCs in primary tumor samples cultured as mammospheres, presented as fold change between hypoxia and normoxia.

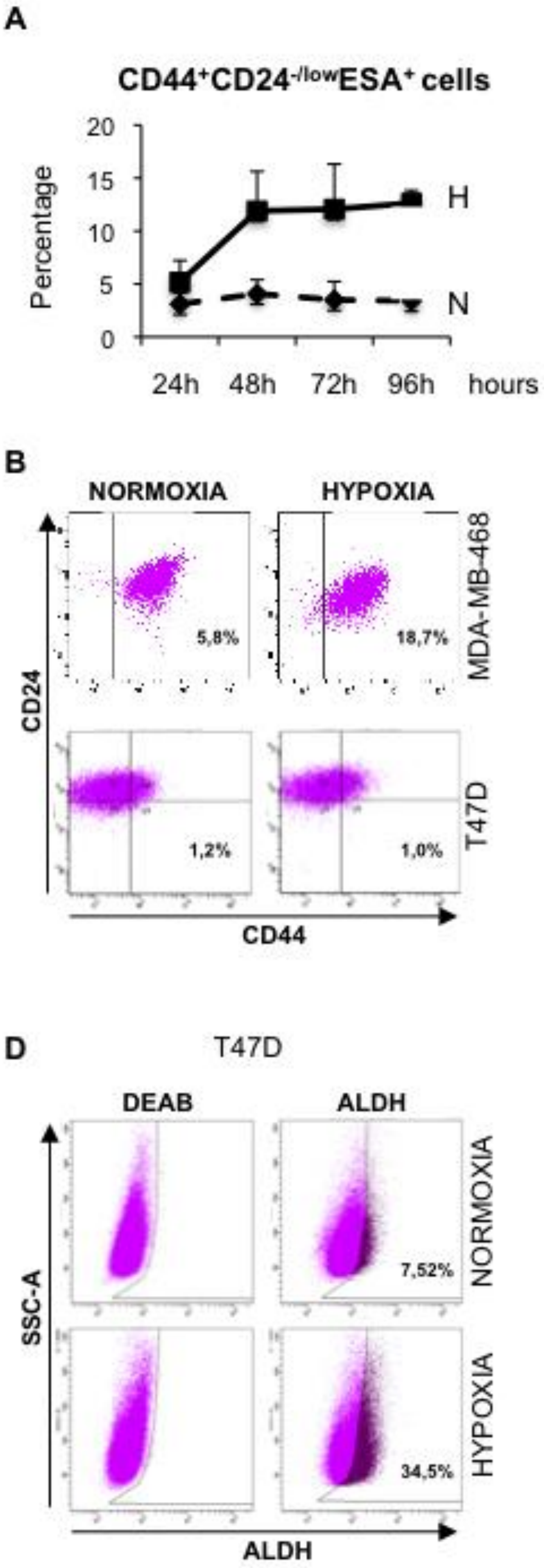
A
CD44+CD24-/lowESA+ cells
Percentage
20
15
10
5
0
H
N
24h
48h
72h
96h
hours
B
NORMOXIA
HYPOXIA
MDA-MB-468
5,8%
18,7%
CD24
T47D
1,2%
1,0%
CD44
C
MCF7
N
H
200 µm
200 µm
D
T47D
DEAB
ALDH
NORMOXIA
HYPOXIA
SSC-A
7,52%
34,5%
ALDH

Supplementary Figure 2. Raw data of FACS analyses using breast cancer cells. (A) Time course of the changes in $CD44^{+}CD24^{-/low}ESA^{+}$ subpopulation of MDA-MB-468 cells cultured in normoxic or hypoxic conditions. B) Representative examples of FACS plots obtained after CD44/CD24 staining in MDA-MB-468 cells and T47D cells grown under different oxygen concentrations. ESA staining is omitted because all cells were ESA^{+} regardless of culture conditions. (C) Representative example of 5-day mammosphere cultures of MCF-7 cells in normoxic (N) or hypoxic (H) conditions. (D) Representative FACS plots of ALDEFLUOR staining performed in T47D cells cultured in normoxia or hypoxia for 3 days.

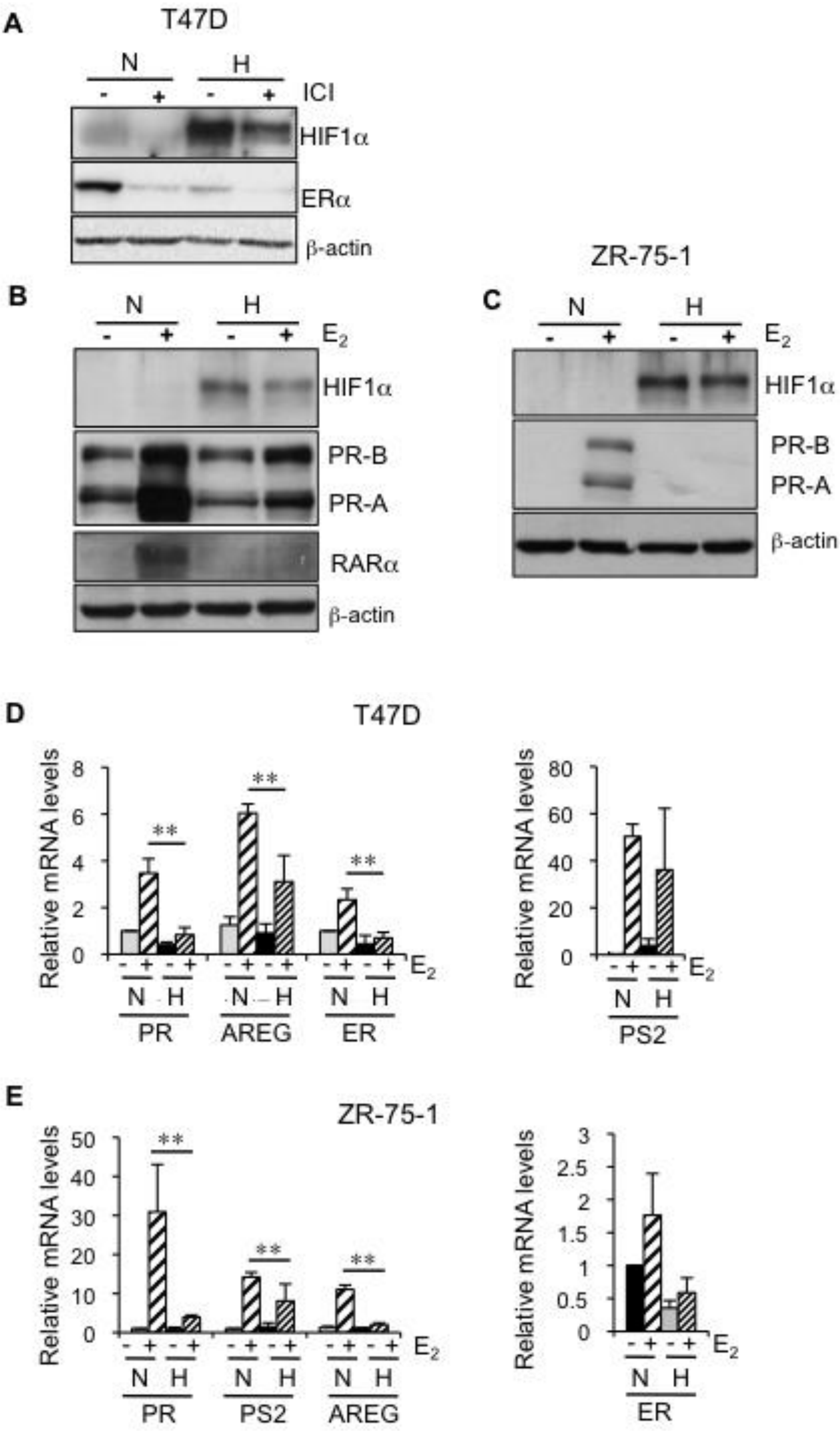
A
T47D
N
H
- + - + ICI
HIF1α
ERα
β-actin
B
N
H
- + - + E_2
HIF1α
PR-B
PR-A
RARα
β-actin
ZR-75-1
C
N
H
- + - + E_2
HIF1α
PR-B
PR-A
β-actin
D
T47D
Relative mRNA levels
8
6
4
2
0
**
**
**
- + - + - + - + - + - + E_2
N H N H N H
PR
AREG
ER
Relative mRNA levels
80
60
40
20
0
- + - + E_2
N H
PS2
E
ZR-75-1
Relative mRNA levels
50
40
30
20
10
0
**
**
**
- + - + - + - + - + - + E_2
N H N H N H
PR
PS2
AREG
Relative mRNA levels
3
2.5
2
1.5
1
0.5
0
- + - + E_2
N H
ER

Supplementary Figure 3. Hypoxia reduces ER expression and transcriptional activity in T47D and ZR75-1 cells. (A) Representative western blots showing expression of HIF1α and ER in T47D cells treated with fulvestrant (ICI 182,870). (B) Representative western blot showing expression of HIF1α, PR and RARα in T47D cells treated or not with 10 nM estrogen, in normoxia or hypoxia. (C) Representative western blot showing expression of HIF1α and PR in ZR75-1 cells treated or not with estrogen, in normoxia or hypoxia. (D, E) mRNA expression levels of ER and its target genes PR, PS2 and AREG in T47D (D) or ZR75-1 (E) cells after culturing them under normoxic or hypoxic conditions, with or without estrogen. Means ±SD of 3 independent experiments are shown. In all cases, cells were starved in estrogen-free conditions for 2 days before treating them with 10 nM estrogen and/or hypoxia for 42 h. $^{**}P<0.01$.

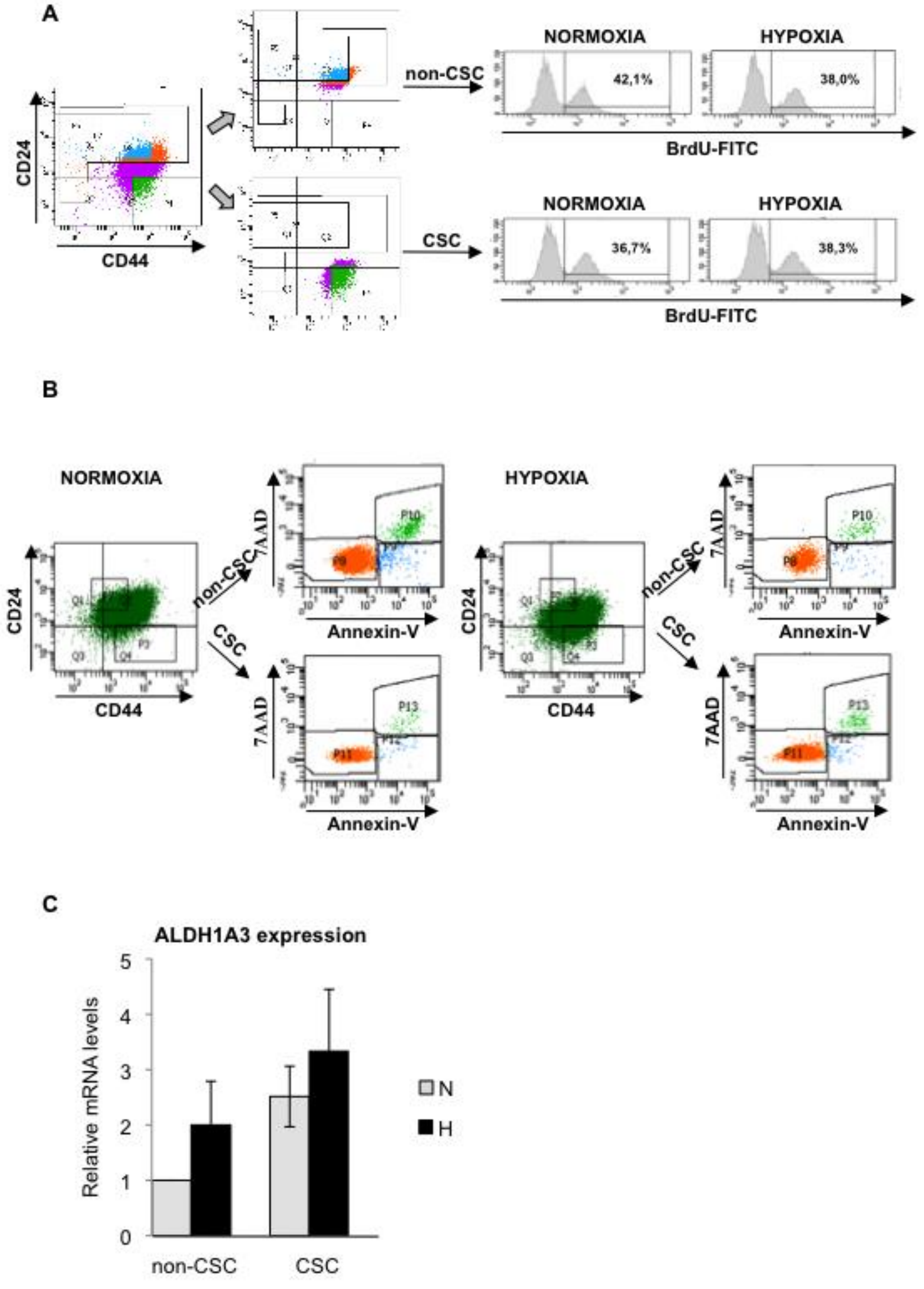

Supplementary Figure 4. Representative plots of proliferation and apoptosis in sorted populations and analysis of ALDH1A3 mRNA expression. (A) Plots representing BrdU

incorporation of sorted CSCs and non-CSCs cultured in normoxic or hypoxic conditions. MDA-MB-468 cells were stained with anti-CD44 and anti-CD24 antibodies, and $CD44^{+}CD24^{-/low}$ CSCs and $CD44^{+}CD24^{high}$ non-CSCs were sorted and cultured in normoxia or hypoxia for 24 h. Cells were then treated with 10 μM BrdU for 1h and harvested. (B) Representative plots of AnnexinV/7AAD stainings. MDA-MB-468 cells cultured in normoxia or hypoxia for 3 days were stained and sorted. $CD44^{+}CD24^{-/low}$ CSCs and $CD44^{+}CD24^{high}$ non-CSCs were then used for annexinV/7AAD staining, in order to detect early and late apoptotic cells. (C) Analysis of ALDH1A3 mRNA expression in T47D cells obtained after culturing sorted $ALDH^{+}$ CSCs and $ALDH^{-}$ non-CSCs in normoxia or hypoxia for 3 days, as seen in Figure 4E and 4F. The figure shows means ±SD of three independent experiments.

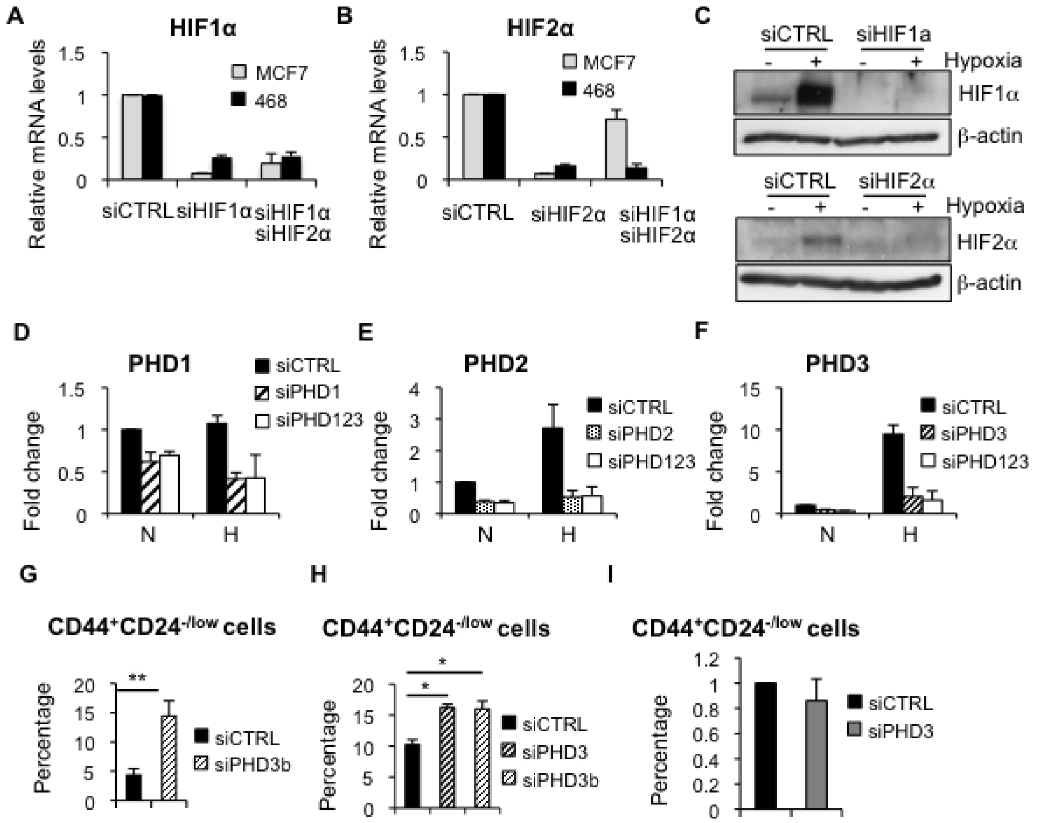

Supplementary Figure 5. Molecular analysis of HIF and PHDs. (A, B) qPCR analysis of silencing efficiencies for HIF1α (A) and HIF2α (B) in MCF-7 or MDA-MD-468 cells, as indicated. (C) Western blot analysis of silencing efficiencies for HIF1α and HIF2α in T47D cells where ALDH activity was evaluated after downregulation of these genes. (D-F) mRNA expression levels of PHD1, PHD2, and PHD3 in experiments where the percentage of $C44^{+}CD24^{-/low}$ CSCs was evaluated after downregulation of these genes. (G) Percentage of $CD44^{+}CD24^{-/low}$ CSCs in MDA-MB-468 cells after downregulation of PHD3 with an alternative siRNA sequence. (H) Percentage of $CD44^{+}CD24^{-/low}$ in BT549 cells after downregulation of PHD3 with 2 different siRNA sequences. (I) Percentage of $CD44^{+}CD24^{-/low}$ in MCF-7 cells after downregulation of PHD3 with a specific siRNA sequence.

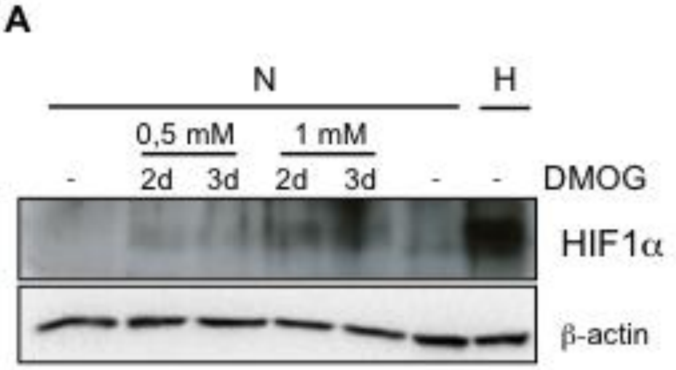

Supplementary Figure 6. Analysis of HIF1α protein expression levels.

(A) Western blot of HIF1α stabilization in MDA-MB-468 cells treated with DMOG or hypoxia.

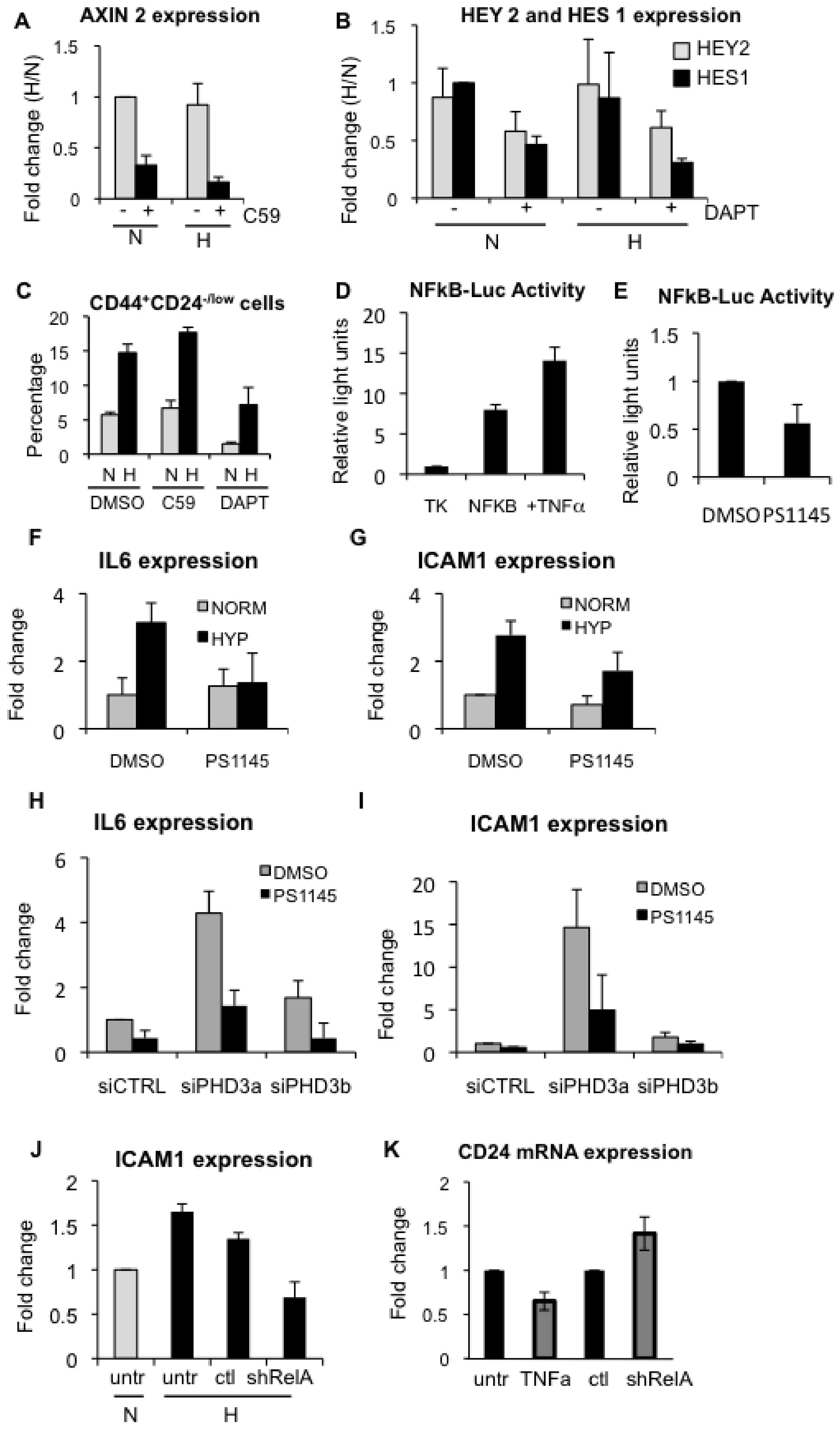
A
AXIN 2 expression
Fold change (H/N)
C59
N
H
B
HEY 2 and HES 1 expression
HEY2
HES1
DAPT
C
CD44+CD24-/low cells
Percentage
DMSO
C59
DAPT
D
NFkB-Luc Activity
Relative light units
TK
NFKB
+TNFα
E
NFkB-Luc Activity
DMSO
PS1145
F
IL6 expression
Fold change
NORM
HYP
G
ICAM1 expression
H
IL6 expression
DMSO
PS1145
siCTRL
siPHD3a
siPHD3b
I
ICAM1 expression
J
ICAM1 expression
untr
ctl
shRelA
K
CD24 mRNA expression
TNFa

Supplementary Figure 7. Analysis of the implication of NFkB signaling in CSCs.

(A) Axin2 mRNA expression levels in MDA-MB-468 cells treated or not with C59 and cultured in normoxia or hypoxia for 3 days. (B) HEY2 (grey bars) and HES1 (black bars) mRNA expression levels in MDA-MB-468 cells treated or not with DAPT and cultured in normoxia or hypoxia for 3 days. (C) Percentage of $CD44^{+}CD24^{-/low}$ in MDA-MB-468 cells cultured in normoxia or hypoxia for 3 days, in the presence, or absence of C59 100 nM (Wnt inhibitor), or DAPT 10 µM (Notch inhibitor). (D) NFkB transcriptional activity in MDA-MB-468 cells stimulated with TNFα 10 ng/ml for 3 h. (E) NFkB transcriptional activity in MDA-MB-468 cells cultured in the presence or absence of 2 µM PS1145. (F, G) IL-6 and ICAM1 mRNA expression levels in MDA-MB-468 cells cultured in normoxia or hypoxia for 3 days, in the presence or absence of 2 µM PS1145. (H, I) IL-6 and ICAM1 mRNA expression levels in MDA-MB-468 cells silenced with siCTRL or siPHD3 for 3 days, in the presence or absence of 2 µM PS1145. Data are presented as fold change with respect to control siRNA in DMSO as 1. (J) ICAM1 mRNA expression levels in BT549 cells untransfected in normoxia (grey bar) or hypoxia (black bars) or transfected with shRelA (as Figure 7H). (K) CD24 mRNA expression levels in BT549 cells untreated (untr) or treated with 10 ng/ml TNFα (TNFa) and transfected with a sh control (ctl) or shRelA/p65 (shRelA).

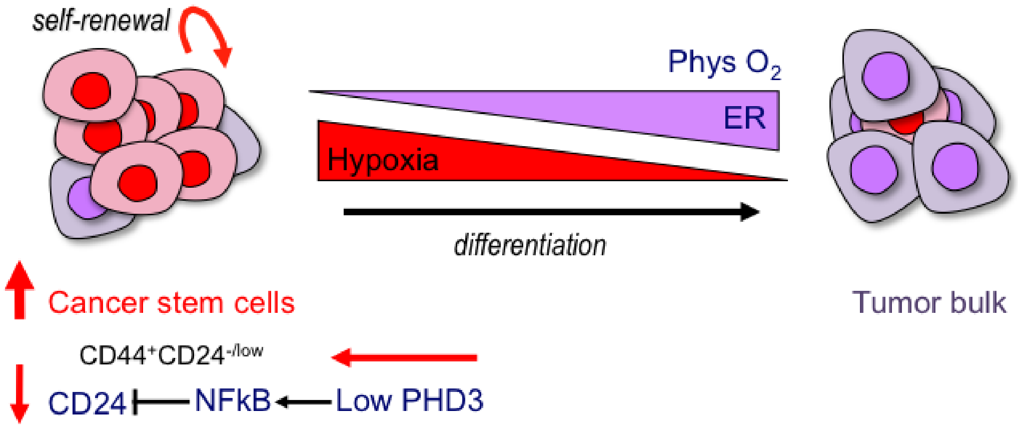

Supplementary Figure 8. Model summarizing the signaling pathways that link hypoxia to dedifferentiation of breast cancer cells.

The increase in CSC following hypoxic stress is at least partly regulated by the NFkB signaling pathway. Hypoxic conditions or down-regulation of PHD3 in ER-negative breast tumor cells leads to activation of NFkB signaling and a reduction in expression of CD24, resulting in the expansion of the CD44$^+$CD24$^{-/low}$ cancer stem cell population.

Supplementary Tables

Supplementary Table 1. Clinical data of patients participating in the study. Samples were obtained from women undergoing reduction mammoplasties with no previous history of breast cancer. NA, not applicable, ND, no data available.

	Age	Day of menstrual cycle	Number of children	Number of children breast-fed	Contraceptive pill intake
**1**	24	21	0	NA	NO
**2**	42	22	1	1	NO
**3**	27	17	0	NA	NO
**4**	43	28	2	2	ND

Supplementary Table 2. Histopathological data of primary tumor samples analyzed for $CD44^{+}CD24^{-/low}ESA^{+}$ content.

	Age	Histol. type	Grade, size and nodal status	ER/PR/HER2	Ki67	p53
1	71	IDC	G2 T2 (2,2) cm N+	ER +++ (98%)<br>PR ++/+++ (82%)<br>HER2 (3+)	+++<br>(16%)	+<br>(15%)
2	55	IDC	G3 T2 N-	ER +++ (80%)<br>PR +++ (80%)<br>HER2 (-1+)	+++<br>(20%)	++<br>(20%)
3	46	IDC	G1, T1c	ER +++ (98%)<br>PR +++ (80%)<br>HER2 (3+)	7%	(<10%)
4	59	IDC	G2, T2	ER +++ (80%)<br>PR +++ (90%)<br>HER2 -	5%	20%
5	68	ILC	G2, T2, N-	ER +++ (80%)<br>PR +++ (80%)<br>HER2 (-)	+++<br>(8%)	-
6	69	Medullar	G3, pT2	ER+++(98%)<br>PR+++(80%)<br>HER2(-)	1-2%	-
7	50	IDC	G2, pT2, pN2, pL1.	ER ++ (40%)<br>PR +++ (70%)<br>HER2 (-)	+++<br>(43%)	unknown
8	51	IDC	G2 T2 2,2cm	ER +++ (96%)<br>PR + (11%)<br>HER2 (2+)	+++<br>(16%)	-
9	79	IDC	G3, T2	ER -<br>PR -<br>HER2 (2+)	+++<br>(60%)	++<br>(80%)
10	53	IDC	G1	ER ++ (50%)<br>PR -<br>HER2 (3+)	++<br>(15%)	-
11	80	IDC	G3 T2 (4cm) N-	ER -<br>PR -<br>HER2 -	+++<br>(80%)	-
12	80	IDC	G3, T1	ER -<br>PR -<br>HER2 (3+)	+++<br>(10%)	-
13	72	C	G3, pT2	ER (-)<br>PR (-)<br>HER2 (-)	+++<br>(80%)	-
14	59	IDC	G2, pT2, pN1a	ER+++ (80%)<br>PR + (11%)<br>HER2 3+	15%	10%
15	36	IDC	G3, pT2, pN0	ER -<br>PR -<br>HER2 (-)	+++<br>(85%)	5%
16	61	ILC	G2, pT2, pN1c	ER ++ +(100%)<br>PR + (11%)<br>HER2 (+)	+<br>(15%)	5%

Supplementary Table 3. Primer sequences used for qPCR amplification of the genes indicated.

Gene	Forward Primer 5' – 3'	Reverse Primer 5' – 3'
36B4	GTGTTCGACAATGGCAGCAT	GACACCCTCCAGGAAGCGA
ERα	CCACCAACCAGTGCACCATT	GGTCTTTTCGTATCCCACCTTTC
PR	CGCGCTCTACCCTGCACTC	TGAATCCGGCCTCAGGTAGTT
AXIN2	CTGGCTCCAGAAGATCACAAAG	ATCTCCTCAAACACCGCTCCA
CD24	TGAAGAACATGTGAGAGGTTTGAC	GAAAACTGAATCTCCATTCCACAA
PS2	TCGGGGTCGCCTTTGGAGCAG	GAGGGCGTGACACCAGGAAAACCA
ICAM1	ACGCTGAGCTCCTCTGCTACTC	GGGCAGGATGACTTTTGAGG
HIF2α	GTCACCAGAACTTGTGC	CAAAGATGCTGTTCATGG
HIF1α	CTGCAACATGGAAGGTATTGCA	TACCCACACTGAGGTTGGTTACTG
PHD1	TGACCGGTTGCTCATTTTCTG	TGGCATAGGCTGGCTTCAC
PHD2	AGCTGGTCAGCCAGAAGAGT	GCCCTCGATCCAGGTGATCT
PHD3	GGCTGGGCAAATACTACGTCAA	CCTGTTCCATTTCCCGGATAG
PS2	TCGGGGTCGCCTTTGGAGCAG	GAGGGCGTGACACCAGGAAAACCA
HES1	TAGCTCGCGGCATTCCAAGC	GTGCTCAGCGCAGCCGTCATCT
HEY2	TTGTTTGTTCCACTGCTGGT	CCCGCCCTTGTCAGTATC
AREG	TGGAAGCAGTAACATGCAAATGTC	GGCTGCTAATGCAATTTTTGATAA
IL6	CCTCGACGGCATCTCAGCCCT	TCTGCCAGTGCCTCTTTGCTGC

Supplementary Table 4. The RNAi sequences used in this study are shown.

Gene	siRNA sequence
siHIF1α	AGGACAAGUCACCACAGGATT
siHIF2α	GCAAAUGUACCCAAUGAUATT
siPHD1	GACAGAAAGGUGUCCAAGUTT
siPHD2	CUUCAGAUUCGGUCGGUATT
siPHD3a	CAGGUUAUGUUCGCCACGUGGTT
siPHD3b	GCAAUGGUGGCUUGCUAUCTT
siCTRL	CCUACAUCCCGAUCGAUGAUGTT
